# Identification and characterization of microRNAs in white and brown alpaca skin

**DOI:** 10.1186/1471-2164-13-555

**Published:** 2012-10-16

**Authors:** Xue Tian, Junbing Jiang, Ruiwen Fan, Haidong Wang, Xiaolin Meng, Xiaoyan He, Junping He, Hongquan Li, Jianjun Geng, Xiuju Yu, Yunfei Song, Danli Zhang, Jianbo Yao, George W Smith, Changsheng Dong

**Affiliations:** 1 College of Animal Science and Technology, Shanxi Agricultural University, Taigu, 030801, People's Republic of China; 2 Division of Animal and Nutritional Sciences, Laboratory of Animal Biotechnology and Genomics, West Virginia University, Morgantown, WV, 26506, USA; 3 Laboratory of Mammalian Reproductive Biology and Genomics, Departments of Animal Science and Physiology, Michigan State University, East Lansing, MI, 48824, USA

**Keywords:** Alpaca, MicroRNAs, Deep sequencing, Coat color, Skin

## Abstract

**Background:**

MicroRNAs (miRNAs) are small, non-coding 21–25 nt RNA molecules that play an important role in regulating gene expression. Little is known about the expression profiles and functions of miRNAs in skin and their role in pigmentation. Alpacas have more than 22 natural coat colors, more than any other fiber producing species. To better understand the role of miRNAs in control of coat color we performed a comprehensive analysis of miRNA expression profiles in skin of white versus brown alpacas.

**Results:**

Two small RNA libraries from white alpaca (WA) and brown alpaca (BA) skin were sequenced with the aid of Illumina sequencing technology. 272 and 267 conserved miRNAs were obtained from the WA and BA skin libraries, respectively. Of these conserved miRNAs, 35 and 13 were more abundant in WA and BA skin, respectively. The targets of these miRNAs were predicted and grouped based on Gene Ontology and KEGG pathway analysis. Many predicted target genes for these miRNAs are involved in the melanogenesis pathway controlling pigmentation. In addition to the conserved miRNAs, we also obtained 22 potentially novel miRNAs from the WA and BA skin libraries.

**Conclusion:**

This study represents the first comprehensive survey of miRNAs expressed in skin of animals of different coat colors by deep sequencing analysis. We discovered a collection of miRNAs that are differentially expressed in WA and BA skin. The results suggest important potential functions of miRNAs in coat color regulation.

## Background

Since the first small RNA (sRNA) – *lin4* was discovered in *C. elegans*, identification of small endogenous non-coding RNA (sncRNA) has received considerable attention. SncRNAs include small interfering RNA [1], miRNA [2] and piwi-interacting RNA, which all regulate genes at the post-transcriptional level [3]. Among these sncRNAs, miRNAs are small, non-coding 21–25 nt RNA molecules that play an important role in regulating gene expression in animals and plants by promoting mRNA degradation and inhibiting mRNA translation [4]. However, other studies revealed that some miRNAs may also function to induce gene expression [5]. Many miRNAs are evolutionary conserved in related species and some even show conservation between invertebrates and vertebrates [6]. Generally, the functions of miRNAs are not limited to the regulation of developmentally timed events. Instead, they have diverse expression patterns and probably regulate many aspects of development and physiology [7]. To date, the miRBase database contains 9072 mature miRNA products from 25 mammalian species. Recently, transcriptome profiling of miRNAs has led to identification of novel miRNAs, provided valuable information on tissue specific expression and evolutionary conservation across species, and served as a cornerstone for subsequent functional analyses.

In adult animals, both hair and skin color depend on pigment produced by melanocytes at the base of the epithelium [8]. Melanocytes in mammals and birds produce two types of melanin, black to brown eumelanin and yellow to reddish brown pheomelanin [9,10]. The quality and ratio of eumelanin to pheomelanin influence the color of skin, fleece and eyes [9]. At present, a large number of genes have been found to affect hair and skin color in humans and other vertebrate species [11-13], although the molecular and cellular mechanisms regulating coat color in fiber-producing species, such as the alpaca, are not completely understood.

Alpacas have a great variety of natural coat colors. Studies of the melanocortin-1 receptor (MC1R) and agouti signaling protein (ASIP) genes in alpacas of different coat colors revealed several polymorphisms that may be predictive of coat color [14-18]. Powell et al. reported that the M87V and S126G variants of MC1R may affect coat color with brown coat color animals showing homozygosity for the methionine and serine alleles [16]. Tyrosinase related protein 1 (TYRP1) is encoded by the brown locus and is involved in eumelanin synthesis [19]. Mutations in *TYRP1* are associated with brown coat color in many species [20,21], but mutations in *TYRP1* associated with brown coat color in alpacas have not been reported to date. Previous studies in our laboratory revealed that the expression of many coat color genes including MC1R, TYRP1, tyrosinase (TYR), tyrosinase related protein 2 (TYRP2), nitric oxide synthase 2 (NOS2), paired box protein 3 (PAX3), and WNT3A was higher in skin of brown versus white alpacas, suggestive of a functional contribution to coat color regulation in this species. However, the potential contribution of miRNAs to differential expression (associated with coat color) of such genes has not been fully elucidated, primarily due to a lack of knowledge of the miRNA transcriptome profile in alpaca skin.

Previous studies have addressed expression and function of miRNAs in mouse, goat and sheep skin [22-25]. For example, miR-203, a skin specific miRNA, was up-regulated in psoriatic plaques and shown to be involved in inflammatory responses and keratinocyte functions [26]. However, studies of the functional role of miRNAs in coat color regulation are limited. Dong et al. over-expressed miR-137 in mice and obtained a series of coat color changes in transgenic animals [27]. Zhu et al. reported differential expression of miR-25 in skin of white versus brown alpacas and a functional role of this miRNA in the regulation of expression of microphthalmia-associated transcription factor (MITF), a transcription factor regulating expression of key genes involved in melanogenesis [28].

To better understand the potential role of miRNAs in the post-transcriptional regulation of genes linked to pigmentation, we have characterized the miRNA transcriptome in skin of alpaca of different coat colors by deep sequencing analysis. We report here the identification of hundreds of conserved miRNAs and candidate novel miRNAs in WA and BA skin. We also provide evidence of differential expression of miRNAs in WA and BA, foundational to future studies of the potential functional role for such miRNAs in post-transcriptional gene regulation linked to coat color.

## Results

### Overall complexity of sRNA pools between the WA and BA skin libraries

**Figure 1:**
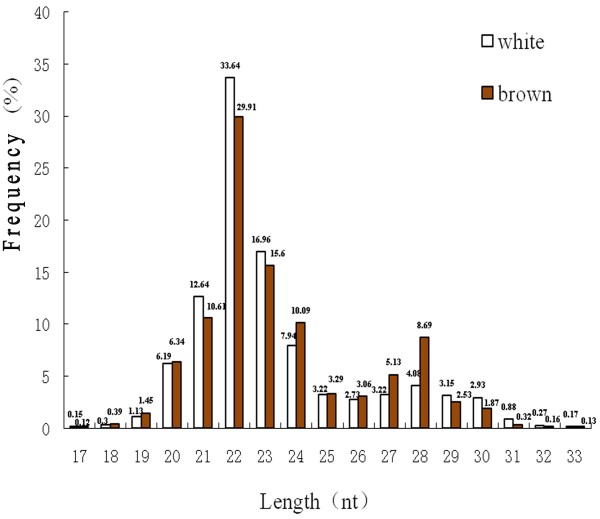
Length distribution and abundance of unique sRNAs in white and brown alpaca skin.

To identify miRNAs expressed in alpaca skin, WA and BA skin small RNA libraries were analyzed by deep sequencing. A total of 17,427,359 and 19,748,517 raw reads were obtained from WA and BA libraries respectively. After filtering out the low quality reads based on base quality value and removing adaptor sequences, 16,834,090 (98.42%) and 19,069,600 (98.49%) clean reads were retrieved, corresponding to 1,058,335 and 977,308 unique sequences, which were the associated counts of the identical sequence reads, respectively. The length distribution of sRNA reads is shown in Figure 1. The majority of the sRNAs were 21–24 nt in length, which is characteristic of sRNAs from metazoans. The most frequent length of sRNA sequenced was 22nt, which is identical to the classical size of Dicer cleavage products.

**Figure 2:**
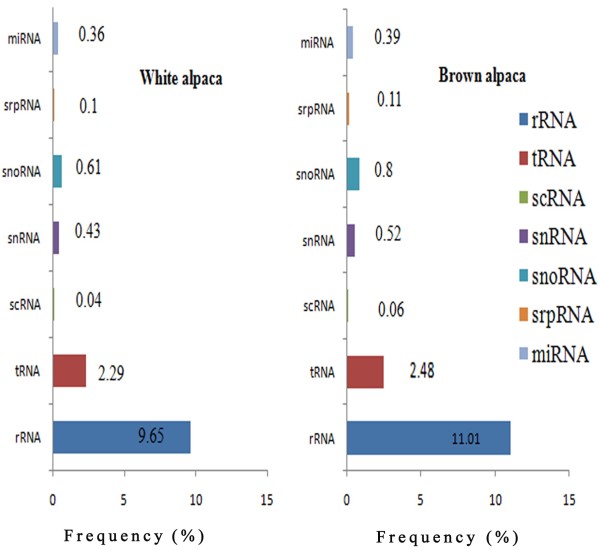
Frequency (%) of different types of sRNA represented in WA and BA skin libraries.

The alpaca genome has been completely sequenced, but at low coverage and is not well annotated. Thus, the alpaca ( http://www.ncbi.nlm.nih.gov) and *Bos taurus* genome databases ( http://www.ncbi.nlm.nih.gov/nuccore/) were combined and used as a reference genome dataset for annotation of alpaca sRNAs. The total sRNA reads were mapped to the combined genome using the SOAP program, yielding 10,389,135 (61.71%) and 12,630,855 (66.24%) genome matched reads in WA and BA libraries, respectively. In order to annotate the classes of all sRNAs, all clean reads were mapped using several databases, including GenBank, Rfam, animal miRNAs (miRBase), exon and intron (*Bos taurus*) and repeat associated RNA. The representations of different types of sRNAs in WA and BA libraries are shown in Figure 2. By aligning the sRNAs to all known animal miRNA precursors, 3820 (6,527,628 total reads) and 3823 (7,583,101 total reads) unique sequences were identified as potentially conserved miRNA in WA and BA skin, respectively.

### Identification of conserved miRNAs in alpaca skin

**Figure 3:**
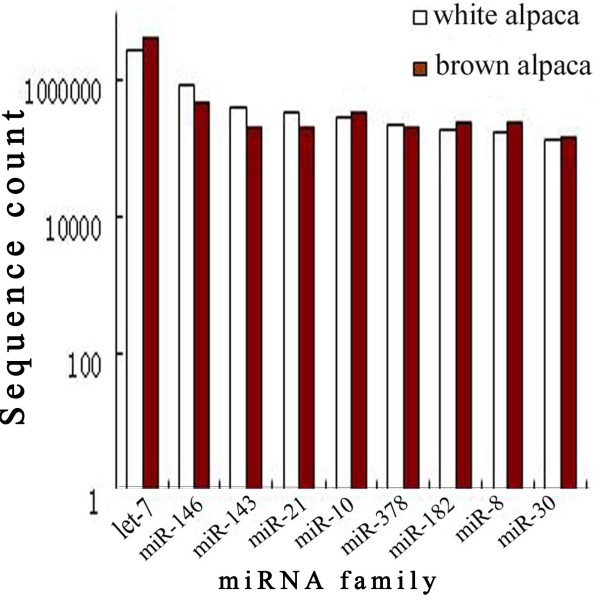
Abundance of conserved miRNAs sequences in white alpaca and brown alpaca.

**Figure 4:**
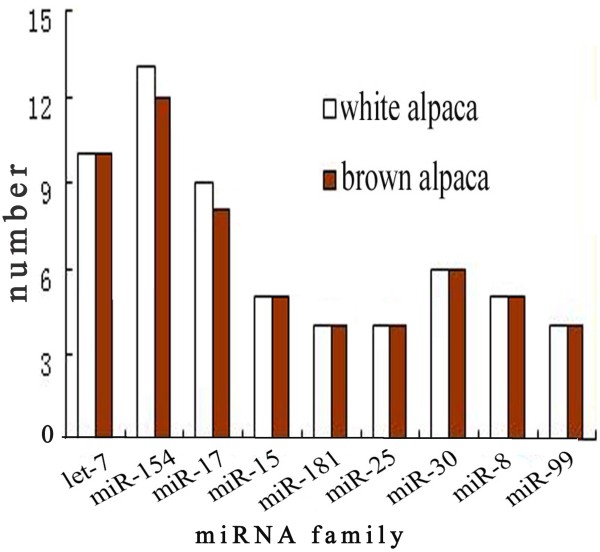
Number of members in different miRNA families in white alpaca and brown alpaca skin.

To further identify conserved miRNAs in alpacas, all sRNA sequences were mapped to known miRNAs in the miRBase 17.0 database. After alignment and additional sequence analysis, 272 and 267 individual candidate miRNAs from WA and BA skin libraries were identified, respectively. Approximately 155 miRNA families were represented in each library. Several miRNA families were represented by over 100,000 reads, such as Let-7, miR-146, miR-143, miR-21, miR-10, miR-378, miR-182, miR-8, and miR-30, which were the 9 most abundant miRNA families represented in WA and BA libraries (Figure 3). The read diversity of miRNA families indicated that different miRNA families have different expression patterns. Besides the diversity in miRNA families, there was considerable variation in number of miRNA family members represented across families. For example, 13 and 12 miR-154 family members were represented in WA and BA libraries, whereas many miRNA families only had 1 to 2 members (Figure 4).

### Differential expression of conserved miRNAs in white and brown alpaca skin

**Figure 5:**
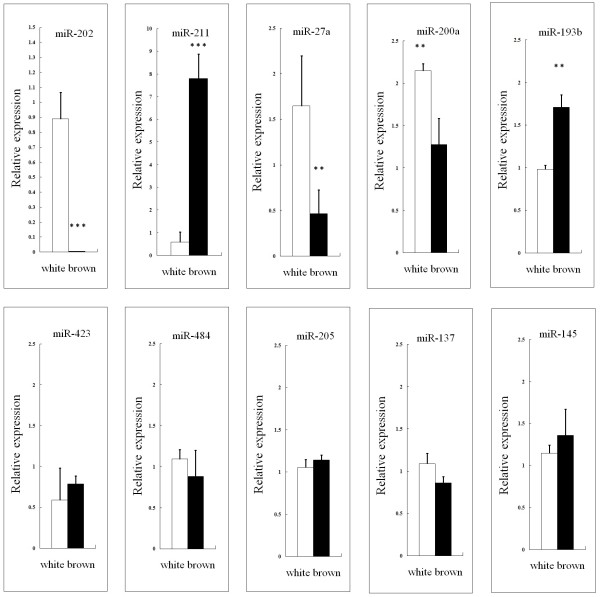
**Real time PCR validation of miRNA expression in skin of alpacas with white vs brown coat color.** Abundance of miR-202, miR-211, miR-27a, miR-200a, miR-193b, miR-423, miR-484, miR-205, miR-137 and miR-145 were normalized relative to abundance of U6 snRNA. Bars in each panel represent the mean ± standard error (n=4 each), ** *P* < 0.05; *** *P* < 0.001.

In high throughput sequencing analysis, the number of reads is reflective of the expression level of miRNAs. Many conserved miRNAs exhibited dissimilar expression patterns in WA versus BA skin. There were 35 miRNAs showing markedly greater abundance in WA skin, and 13 miRNAs more highly expressed in BA skin (Table 1). For example, miR-202, miR-542-5p, miR-424, miR-370 and miR-22-3p were more abundantly expressed in WA skin, while miR-211, miR-184, miR-486, miR-885 and miR-451 were more highly expressed in BA skin. In order to validate the deep sequencing results, 10 miRNAs were selected to determine their expression levels in white and brown alpaca skin by real time PCR. The results of real time PCR analysis confirmed the deep sequencing results (Figure 5). For example, based on deep sequencing results, the expression level of miR-211 in WA was almost 20 times higher than observed in BA. Based on real time PCR results, the expression level of miR-211 in WA skin was also 13 times higher than observed in BA skin.

**Table 1:** Significantly expressed miRNAs in white and brown alpaca skin

**miR-name**	**NEL in brown**	**NEL in white**	**FC**
**White alpaca**			
miR-202	1.9927	95.2234	47.7861
miR-424*	0.1573	1.6633	10.5741
miR-542-5p	0.3146	2.8514	9.0636
miR-424	3.2512	25.1870	7.7470
miR-370	0.5244	3.6236	6.9100
miR-22-3p	207.9226	1354.8698	6.5162
miR-27a-3p	71.6323	429.4856	5.9957
miR-450	2.0976	12.0589	5.7489
miR-34c	11.3794	56.1955	4.9383
miR-122	5.9781	28.0977	4.7001
miR-136	0.3146	1.2475	3.9653
miR-493	0.8915	3.2672	3.6648
miR-216a	2.9366	10.0986	3.4389
miR-671	2.0976	6.8908	3.2851
miR-494	1.0488	3.0890	2.9452
miR-190a	4.2476	11.7024	2.7551
miR-18a	4.2476	11.5836	2.7271
miR-124a	0.7866	2.1385	2.7187
miR-124b	0.7866	2.1385	2.7187
miR-148a	1653.0499	4265.9865	2.5807
miR-206	2.7269	6.8908	2.5270
miR-204	1.8354	4.6335	2.5245
miR-369-3p	3.4610	8.7323	2.5231
miR-345-3p	1.6256	4.0394	2.4849
miR-193a-3p	0.5768	1.4257	2.4718
miR-874	0.8915	2.1385	2.3988
miR-19a	2.3073	5.4057	2.3429
miR-143	10818.3706	24308.7093	2.2461
miR-1296	1.4683	3.2672	2.2252
miR-210	3.6708	7.9006	2.1523
miR-301a	0.839	1.7821	2.1241
miR-27a-5p	55.009	112.8662	2.0518
miR-146b	25987.7501	53201.0343	2.0472
miR-200a	79.708	162.1709	2.0346
miR-17-3p	3.9854	8.0788	2.0271
**Brown alpaca**			
miR-211	47.4053	2.9108	16.2860
miR-184	232.0447	22.2762	10.4167
miR-486	983.9221	151.6565	6.4878
miR-885	5.1915	0.9505	5.4619
miR-451	1122.4147	411.0706	2.7305
miR-133a	4.1427	1.6039	2.5829
miR-299	1.7829	0.7128	2.5013
miR-23a	708.5623	304.5606	2.3265
miR-25	504.2581	226.8017	2.2233
miR-23b-3p	823.9292	371.211	2.2196
miR-1224	6.6598	3.0296	2.1982
miR-15b	121.5547	58.5716	2.0753
miR-193b	207.1360	100.4509	2.0620

Nel, normalized expression level of miRNA in a sample; FC, fold change of miRNAs between white and brown alpaca skin; Nel = actual miRNA count/total count of clean reads×1000000.

### Sequence divergence within known miRNAs

**Figure 6:**
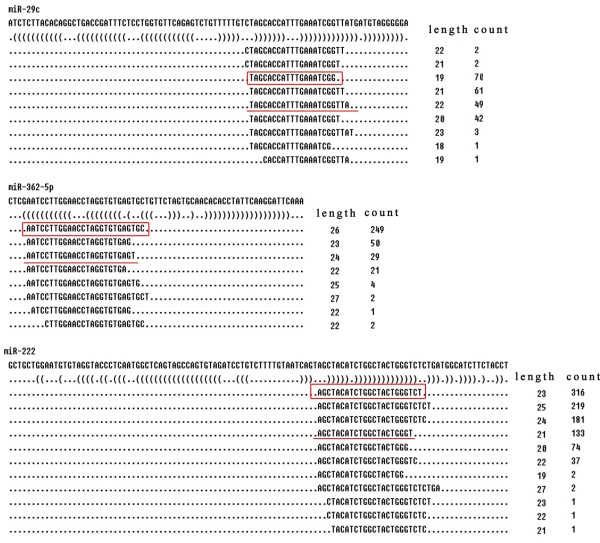
**Heterogeneity at the 5’ and 3’ ends of the sequenced reads.** Pre-miRNAs, structures, and multiple isoforms of expressed mature miR-29c, miR-362-5p, miR-222 sequences and their read counts are shown. The red underline sequences are annotated in miRBase17.0. The boxed sequences owned the highest read counts.

**Figure 7:**
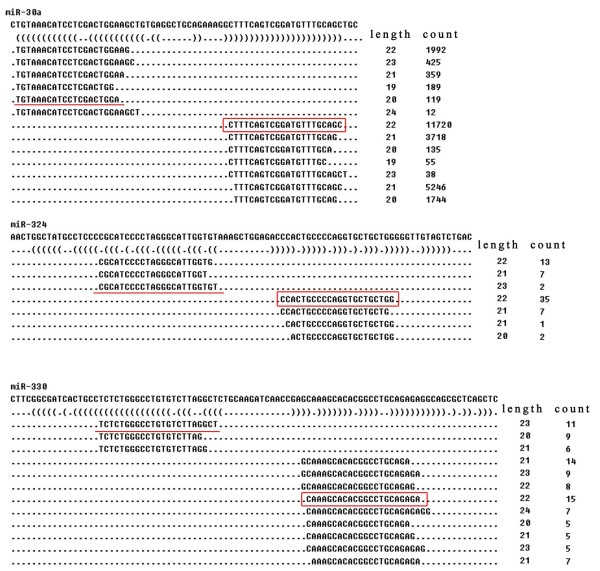
**Annotated miRNA and maximal sequences from different arms of the precursors.** The red underlined sequences are the annotated mature sequences in miRBase while the boxed sequences represent the maximum counts in sequencing database.

The heterogeneity across sequenced reads at the 5’ and 3’ ends from the same arms within individual known miRNAs detected were also analyzed. The 5’ ends exhibited less heterogeneity than the 3’ ends. For instance, miR-29c, miR-362-5p, and miR-222 were highly represented and sequenced from the two libraries. The predominant reads for these miRNAs obtained from deep sequencing data varied from the mature miRNA sequences in miRBase at the 3’ end, and they should be regarded as the primary functional molecule (Figure 6). Collectively from the two libraries, the 5’ and 3’ end of 109 alpaca miRNAs were different in sequence from corresponding annotated miRNAs for each species in miRBase (Additional file 1). Furthermore, several abundant miRNAs with higher sequencing reads, like miR-30a, miR-324 and miR-330, were derived from different arms of the hairpin precursor than corresponding annotated miRNAs in miRBase (Figure 7). Thus, results suggest that the precursors of these miRNAs develop functional molecules from both arms in the alpaca.

### Target gene prediction for miRNAs in alpaca

MiRNAs have functions in numerous developmental, physiological and pathological processes [29]. In order to provide insight into potential biological roles of miRNAs identified in alpaca skin, target prediction analysis was performed for alpaca skin miRNAs identified. The rules employed for target prediction were based on those reported by Allen et al. [30] and Schwab et al. [31]. There were 6317 genes predicted as the putative targets of conserved miRNAs in WA (272 miRNAs) and BA (267 miRNAs) skin, respectively. The predicted target genes were then subjected to gene ontology analysis for functional classification. Although the target functions of miRNA in WA and BA skin had some divergence, the top functions of targets were binding, transferase activity, molecular transducer activity, and receptor activity (Additional file 2). The KEGG pathway was also used to further classify putative miRNA targets. All predicted targets were clustered into 257 pathways in WA and BA, respectively. Results were then interrogated for potential target genes implicated in coat color regulation and the melanogenesis pathway. Many genes in the melanogenesis pathway have already been shown to have a relationship with coat color in alpaca [32-35], such as pro-opiomelanocortin (POMC), melanocyte-stimulating hormone (MSH), *MITF*, kit oncogene (KIT) and *TYR*. Moreover, several miRNAs whose targets are involved in melanogenesis were expressed differentially in WA and BA skin. The target genes for the top 4 significantly expressed miRNAs in WA and BA included in the melanogenesis pathway are shown in Table 2.

**Table 2:** Targets for top 4 significantly expressed miRNAs in white and brown alpaca involved in melanogenesis

**Name**	**Predicted targets involved in melanogenesis**
miR-211	CPNE8, PRKCG, TMEM135, ARMC8, DCT, PLCB1,WNT3
miR-424*	GNAI2, TCF7, CREB3, FZD7, MYOF, PRKACB, KIT, GNAI1, PLCB1, SYT11, SYT1, MCTP1, FZD1, SYT4, CALM
miR-202	CPNE8,TCF7, KRAS, WNT7A, PRKCB, KIT, CAMK2D, CABP2, EDNRB, WNT5A, FZD4
miR-184	CAMK2A, FAM193B, MYOF, ADCY5, ADCY8, PRKCB, PLCB3, RAF1, SYT1, WNT11, PRKACA, WNT16, PLCB2

### Identification of novel miRNAs in alpaca skin

Because the alpaca genome has not been annotated, the bovine genome was utilized to identify potential novel miRNAs. It is well known that the important feature of miRNAs distinguishing them from other small RNAs is the ability of their genomic flanking sequences to fold into a hairpin structure [36]. The Mireap software was used to predict novel miRNAs using these properties. After filtering, 88 and 94 sequencing reads from WA and BA skin remained. Blast analysis against all mature miRNAs in miRBase, revealed 55 miRNAs from WA and 63 miRNAs from BA libraries that had no homology with other species. In these novel miRNAs, 31 miRNAs were common between WA and BA libraries. Thus, a total of 87 unique novel miRNAs were identified (Additional file 3). The hairpin structures for these novel miRNAs were denoted in Additional file 4. The lengths of these novel miRNAs ranged from 21 to 24nt, and the minimum free energy (MFE) of the novel miRNAs varied from −55.90 to −19.00 kcal mol^-1^. The average MFE of novel miRNAs was −34.83 kcal mol^-1^, which is lower than tRNA and rRNA*.* This result was similar to results of Bonnet et al. [37] indicating that the majority of miRNAs exhibit a folding free energy which is lower than shuffled sequences. To further identify genuine miRNAs, the length of the RNAs was also considered. The MFE index (MFEI) was used to re-evaluate the potential novel miRNAs identified. Zhang et al. suggested that the MFEI for miRNAs was 0.97, significantly higher than tRNAs, rRNAs or mRNAs [38]. The MFEI of the 87 potential novel miRNAs ranged from 0.38 to 2.45. Using the MFEI cutoff value of 0.97, 22 novel miRNAs present in the two libraries were identified (Table 3). Expression of these new miRNAs was typically several to a hundred counts lower than conserved miRNAs.

**Table 3:** Novel miRNAs identified in alpaca skin

**Name**	**Sequence**	**MFEI**
Alpaca-novel-82	TCGCTCTCCTGCTCGCTCTGC	0.9789
Alpaca-novel-60	TCTGGAGGACGCCGCTCGCGCTC	0.9811
Alpaca-novel-56	GAATGGTGCTCCCTGGAATTGT	0.9974
Alpaca-novel-24	AGGGAACCTTGAAAAGCTGAAG	0.9986
Alpaca-novel-83	CAGGACCTGGGGACACCATTGT	1.0079
Alpaca-novel-37	TGTTTCTCAGAAGACTGTAGT	1.0155
Alpaca-novel-17	TGAACGGGGCCCTTCTGGTAG	1.0246
Alpaca-novel-61	CCTCACCTGTCATTCTCCCAGA	1.0324
Alpaca-novel-31	ATTGGCATGTCCTGGAATGAG	1.0554
Alpaca-novel-53	CCAAACCAGTTGTGCCTGTAG	1.0602
Alpaca-novel-45	ACTTTGGATTTGAGTCTCTGGT	1.1026
Alpaca-novel-8	ACAAAGTTGGCTGCCTGTGAGC	1.1197
Alpaca-novel-9	TTGGTTGAATACGTGGATGTGG	1.1877
Alpaca-novel-33	ATCCGTGGTTGGTTGAATATGC	1.2365
Alpaca-novel-32	TGAATGGCACCTTATGAGTAGA	1.2418
Alpaca-novel-76	AACTCAGTGTCAGATAGGAAGA	1.2434
Alpaca-novel-27	TTAGTGATGTTGGTTAAAAGAG	1.3272
Alpaca-novel-44	TCTGAAATTTAAATGTAACCGG	1.3920
Alpaca-novel-43	TTGTTGTTGGTTGAATAGTATT	1.5895
Alpaca-novel-68	TAATAGGTGATCAAATGAATGA	1.9358
Alpaca-novel-81	ATTGATCTTTGACTATAACTG	2.0412
Alpaca-novel-8	ACAAAGTTGGCTGCCTGTGAGC	2.4490

MFEI, MFE index.

## Discussion

Studies on expression and function of miRNAs in evolutionarily diverse agricultural species are limited. The alpaca is a domestic mammal specialized in fiber production [39] and has more than 22 natural coat colors [40-44]. In this report, we explored the miRNA transcriptome in alpaca skin using deep sequencing technology, and provided a foundation for future functional studies on the relationship between miRNA expression and development and function of skin including coat color.

Many miRNAs have been identified by traditional Sanger sequencing and microarray methods. However, the high throughout-sequencing method which yields 400,000 reads per run and identifies low abundance tags is a much more comprehensive method for miRNA profiling [45]. Using this sequencing approach, we found 22 novel, as well as 272 and 267 conserved miRNAs in WA and BA skin libraries [2]. According to the new miRNA family classifications in Rfam (miRBase 17.0), 272 miRNAs from WA and 267 miRNAs from BA were grouped into 155 families. Most of these identified miRNA families are also conserved in other mammalian species, such as mouse [23,46], human [26], goat and sheep [22]. Expression of these conserved alpaca miRNA families differed between WA and BA skin, based on both abundance and representation within miRNA families. For example, miR-146 and miR-8 families both had abundant reads in WA and BA skin libraries, but miR-146b (miR-146 family) and miR-141 (miR-8 family) were of greater abundance in WA than BA. This significant variation maybe attributed to the timing and location of miRNA expression during skin development in adult alpacas. In many miRNA families, there was a predominant member whose reads were significantly higher than other members. For example, the Let-7 miRNA family contains 10 members. The reads for let-7a were more than one million, but let-7i only had five thousand reads. It is possible that the predominant member in each miRNA was responsible for the regulatory role at the developmental stage in which skin samples were collected in the present studies.

We compared the expression of conserved miRNAs between WA and BA skin and identified differentially expressed miRNAs from the two libraries. The expression of miRNAs has an established relationship with cell proliferation and differentiation. As such, differences in miRNA expression between animals possibly reflect alterations in growth and differentiation. Heterochrony, or change in the timing of developmental growth, is known to alter the overall shape of developing tissues, leading to differences in physiology [47]. Alternatively, there are pronounced phenotypic differences between WA and BA which include fiber diameter, staple length, hair color and other hair characteristics [48,49]. Thus, differences in miRNA abundance may also be related to hair color and other fiber characteristics. The functional role of such differentially expressed miRNAs is the focus of future investigation.

In animals, miRNAs target mRNAs with partial sequence complementarity [50]. One miRNA is inferred to influence expression of hundreds of mRNAs via this flexible recognition. There were many differentially expressed miRNAs identified between WA and BA skin, so the next step was to search for putative specific genes targeted by these miRNAs. The major physiological difference of interest between the samples analyzed was coat color, so we focused on potential target genes regulating pigmentation. Based on KEGG pathway analysis, we found 117 potential miRNA target genes involved in melanogenesis. Many miRNAs targeting these genes have been reported. For examples, miR-137 [51] and miR-25 [28] were previously shown to regulate the expression of MITF, which is an important regulator of melanocyte growth, maturation, apoptosis and melanogenesis. In the present study, miR-137 and miR-25 were found in both libraries and their target genes also included MITF. Haflidadóttir et al. showed that miR-148 affects Mitf mRNA expression in melanoma cells through a conserved binding site in the 3’-UTR sequences of mouse and human Mitf [52]. In our studies, miR-148 showed greater expression in WA versus BA. So, it is possible that miR-148 may contribute to coat color differences between WA and BA.

The present studies also revealed that both miR-193a and miR-193b are differentially expressed in WA and BA skin. Gao et al. reported that Kit expression was subject to post-transcriptional regulation by miR-193a and miR-193b in human acute myeloid leukemia [53,54]. In humans, Kit contributes to the regulation of skin pigmentation [55] and we have previously reported Kit expression is higher in skin of BA versus WA [34]. Thus, miR-193a and miR-193b may play a role in coat color determination in alpacas via regulating the expression of Kit.

## Conclusions

Studies reported provide novel information on miRNA expression in alpaca species, including conserved miRNAs and potential novel miRNAs, and insight into differential miRNA expression associated with coat color and respective target genes. The predicted targets of the conserved miRNAs include 117 genes related to melanogenesis. Further validation of the functional role of differentially expressed miRNAs and their targets will provide better understanding of the miRNA regulatory mechanisms influencing coat color.

## Methods

### Sample collection and total RNA extraction

Housing and care of alpacas and collection of skin samples for use in described experiments were conducted in accordance with the International Guiding Principles for Biomedical Research Involving Animals ( http://www.cioms.ch/frame 1985 texts of guidelines. html). Three white and three brown healthy adult alpaca (*lama paco*) were selected from the alpaca farm in Shanxi Agriculture University (Shanxi, China) for sample collection. Three pieces of skin (2cm ×3 cm) from the hindquarter were collected under local anesthesia and immediately stored in liquid nitrogen. Total RNA from the samples was extracted using Trizol reagent (Invitrogen, Carlsbad, CA, USA) according to the manufacturer’s instructions. The RNA integrity was evaluated by gel electrophoresis and the RNA concentration was measured by OD260 reading using a Nanodrop spectrophotometer.

### Small RNA library preparation and sequencing

For deep sequencing, total RNA samples were size-fractionated on a 15% PAGE gel, and the 16–30 nt fraction was collected. The 5’RNA adaptor and 3’RNA adaptor were ligated to the RNA pools, and the RNAs of 64–99 nts were isolated through gel elution and ethanol precipitation. PCR products were purified and small RNA libraries were sequenced using Illumina Genome Analyzer. Sequencing was carried out at the ShenZheng Genomics Institute (BGI, China).

### Initial processing of reads and identification of conserved miRNAs

Raw sequence reads were produced by the Illumina HiSeq2000 and processed into clean full length reads by the BGI small RNA pipeline. During the procedure, all low quality reads, including 3’ and 5’ adapter contaminants were removed. The remaining high quality sequences larger than 30nt and smaller than 18nt were discarded. Then, all high quality sequences were mapped to *Vicugna pacos* genome ( http://www.ncbi.nlm.nih.gov/nuccore/206581138) and *Bos Taurus* genome ( http://hgdownload.cse.ucsc.edu/goldenPath/bosTau4/bigZips/bosTau4.fa.gz) by SOAP [56]. All clean reads were compared with the sequences of non-coding RNA (rRNA, tRNA, snRNA, snoRNA) available in Rfam ( http://www.sanger.ac.uk/software/Rfam) [57] and the GenBank non-coding RNA database ( http://www.ncbi.nlm.nih.gov/) to classify degradation fragments of non-coding RNA. We followed the rule [58]: rRNAetc, in which Genbank > Rfam> known miRNA > repeat > exon > intron. Subsequently, the remaining sequences were aligned to the whole animal miRNAs deposited in miRBase 17.0 to obtain the known miRNAs in alpaca as well as base bias at the first position of identified miRNAs and at each position of all identified miRNAs respectively. If a known miRNA in alpaca showed homology with less than two mismatches (or >90% homology), it was considered as evolutionary conserved.

### Validation of conserved miRNA expression

Ten conserved miRNAs were selected for validation by quantitative real time PCR analysis. Total RNA was isolated from eight alpaca skin samples (4 brown, 4 white). One μg of DNase-treated RNA was converted to cDNA using specific stem-loop RT primer (Additional file 5) and HiFi-MMLV cDNA kit mix (CWBIO, Beijing, China). The cDNA was then used for real time PCR quantification of miRNAs using miRNA specific primers (Additional file 5) in combination with the universal primer. U6 snRNA was used as an endogenous control (primers listed in Additional file 5). Quantitative real-time PCR was performed in triplicate on the Stratagene Mx3005P system. The 25 μL PCR reaction included 12.5μL SYBR *Premix Ex Taq*^TM^ II (TaKaRa, Dalian, China), 1.0 μL specific forward primer, 1μL universal primer, 0.5 μL ROX reference dye, 2.5 μL diluted (4 times) cDNA and 7.5 μL water. Cycling parameters were 95°C for 30 sec followed by 40 cycles of 95°C for 5 sec, 56 °C or 58 °C for 20 sec and 72 °C for 15 sec. Melting curve analyses were performed following amplifications. Quantification of selected miRNA transcript abundance was performed using the comparative threshold cycle (CT) method established by Livak et al. [59]. The abundance of selected miRNAs was normalized relative to that of U6 snRNA [60].

### Identification of novel miRNAs

The prediction of alpaca candidate novel miRNAs was conducted using criteria that were previously developed for animal miRNA prediction [61]. The criteria were as follows: the putative mature sequence must reside at the stem region and its size was limited to 20-24nt; the frequency of putative mature sequence should not be below 5; the folding free energy of the stem-loop structure was limited to below −18 kcal mol^-1^; the maximum tolerance of a bugle size was 4 nucleotides; the maximum size of difference between miRNA and miRNA* was 5 nucleotides and 35 nucleotides; the sequence asymmetry between miRNA and miRNA* could not exceed 5 nucleotides. The selected sequences were then folded into a secondary structure using mFold3.2 ( http://mfold.rit.albany.edu). If a perfect stem-loop structure was formed, the small RNA sequence was located at one arm of the stem and met the above criteria, the small RNA was then considered as a candidate novel alpaca miRNA. The formula for calculating MFEI was: MFEI = [(MFE / length of the RNA sequence) ×100] / (G + C )%].

### Target gene prediction

Mireap software ( https://sourceforge.net/projects/mireap/) was used to predict the target genes for conserved miRNAs identified. The criteria were as follows: no more than four mismatches between miRNA & target (G-U bases count as 0.5 mismatches); no more than two adjacent mismatches in positions 2–12 of the miRNA/target duplex (5^′^ of miRNA); no mismatches in positions 10–11 of miRNA/target duplex; no more than 2.5 mismatches in positions 1–12 of the miRNA/target duplex (5^′^ of miRNA); MFE of the miRNA/target duplex should be >75% of the miRNA bound to its perfect complement.

## Abbreviations

miRNAs: MicroRNAs; WA: White alpaca; BA: Brown alpaca; sRNA: Small RNA; sncRNA: Small endogenous non-coding RNA; MC1R: Melanocortin-1 receptor; ASIP: Agouti signaling protein; TYRP1: Tyrosinase related protein 1; TYR: Tyrosinase; TYRP2: Tyrosinase related protein 2; NOS2: Nitric oxide synthase 2; PAX3: Paired box protein 3; MFE: Minimum free energy; MFEI: MFE index; MITF: Microphthalmia-associated transcription factor; POMC: Pro-opiomelanocortin; MSH: Melanocyte-stimulating hormones; KIT: Kit oncogene.

## Competing interests

The authors declare that they have no competing interests.

## Authors' contributions

XT designed the study, performed the analysis, analyzed the data and wrote the paper. JBY and GWS participated in the design of the study, analyzed the data and critically revised the manuscript. DLZ and YFS took part in collecting samples and the data analysis. JBJ, RWF, HDW, XLM, XYH, JPH, HQL, JJG and XJY conceived of the study and critically revised the manuscript. CSD participated in the design of the study, analyzed the data and participated in writing. All authors read and approved the final manuscript.

## Supplementary Material

Additional file 1 **Table S1.** Heterogeneity at the 5’ and 3’ ends of the sequenced reads. The list of alpaca miRNAs different with miRNAs in miRBase based on sequencing counts. Click here for file

Additional file 2 **Table S2.** Gene Ontology analysis of miRNAs targets. Targets of conserved miRNAs were grouped by Gene Ontology. Click here for file

Additional file 3 **Table S3.** Novel miRNAs in white and brown alpaca skin. Sequences and MFE of candidate novel miRNAs in white and brown alpaca. Click here for file

Additional file 4 **Structure S4.** Stem–loop structure of novel miRNAs. The stem-loop structure of 87 novel miRNAs in white alpaca and brown alpaca. The red underlined sequences are the predicted mature sequences. Click here for file

Additional file 5 **Table S5.** miRNAs primers for real time PCR. List of miR-202, miR-211, miR-27a, miR-200a, miR-193b, miR-423 miR-484, miR-205, miR-137, miR-145 and U6 specific stem-loop RT primer, specific primers and universal primer. Click here for file
