# Supplementary material for: Identification and characterization of microRNAs in white and brown alpaca skin
Source: BMC Genomics. 2012 Oct 16;13:555. doi: 10.1186/1471-2164-13-555 (PMC3508611; doi:10.1186/1471-2164-13-555)

Alpaca-novel-1

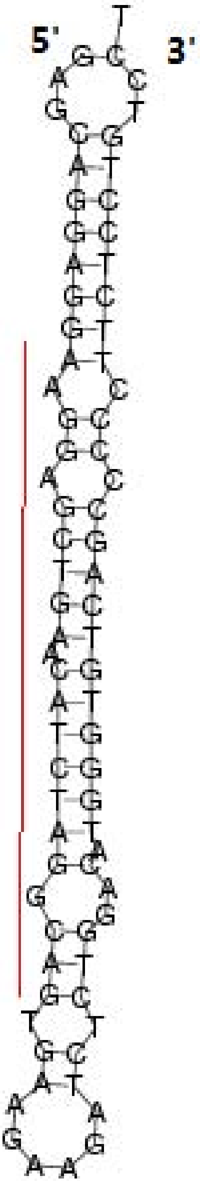

Alpaca-novel-2

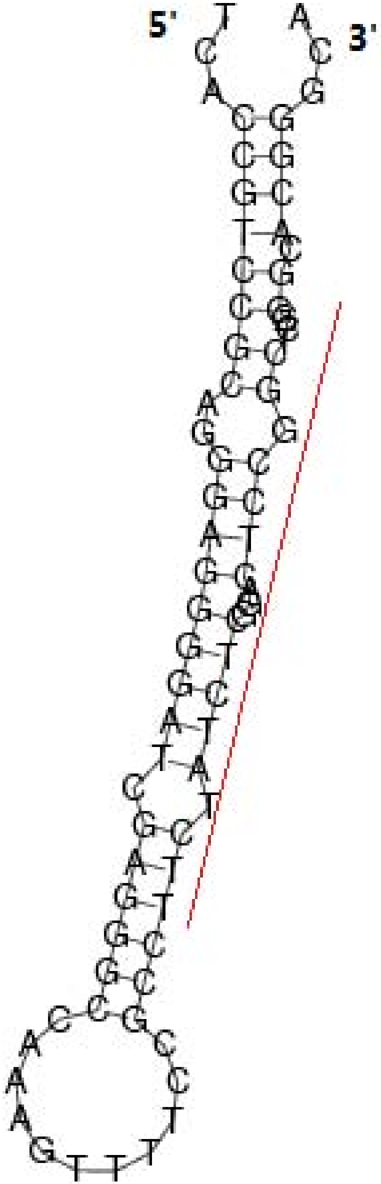

Alpaca-novel-3

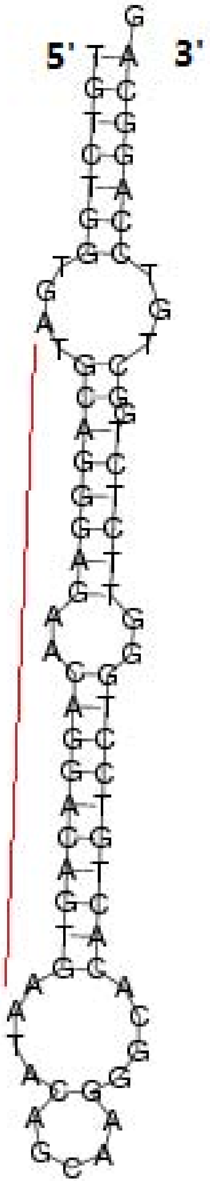

Alpaca-novel-4

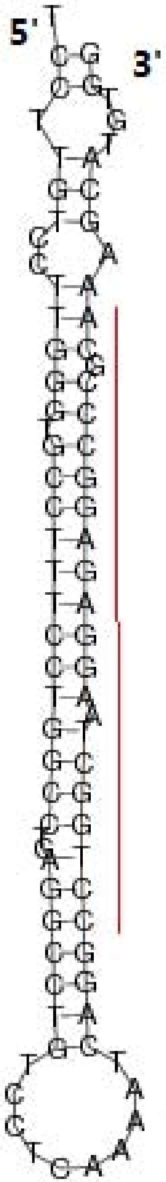

Alpaca-novel-5

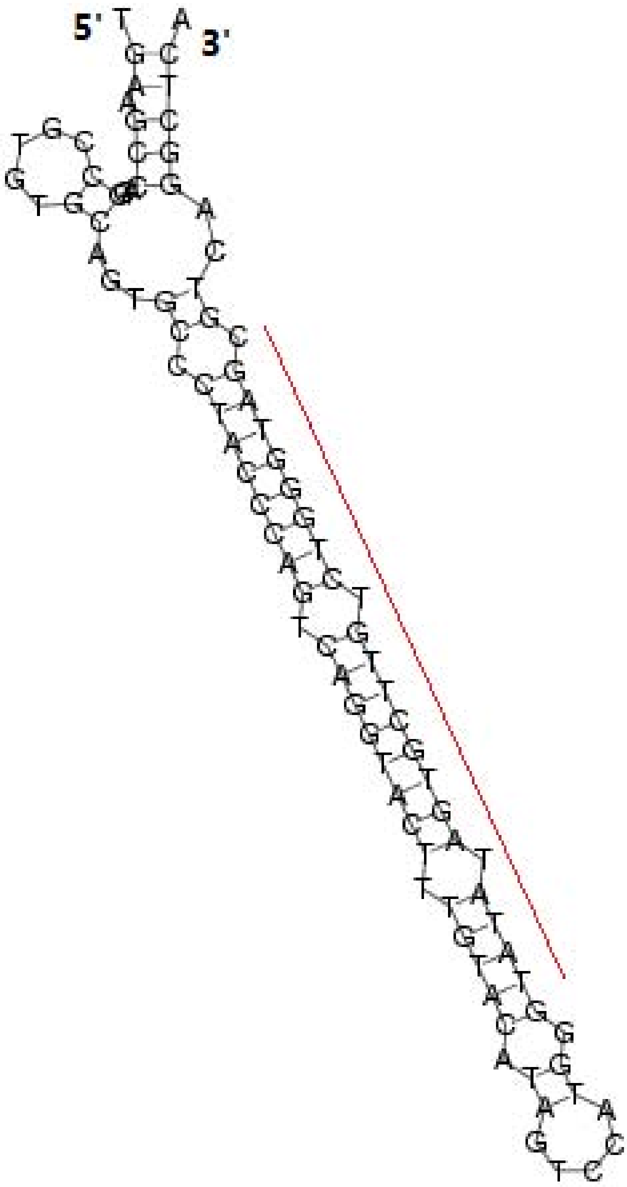

Alpaca-novel-6

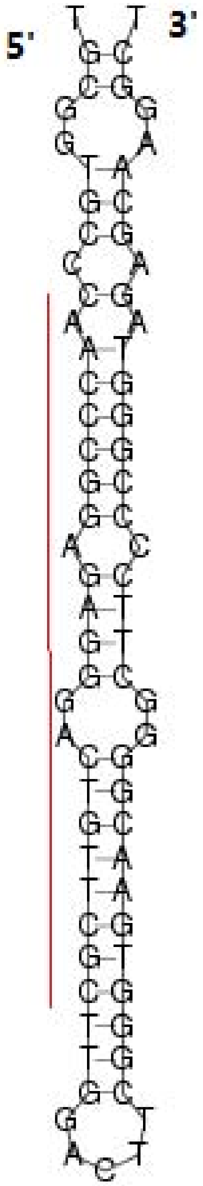

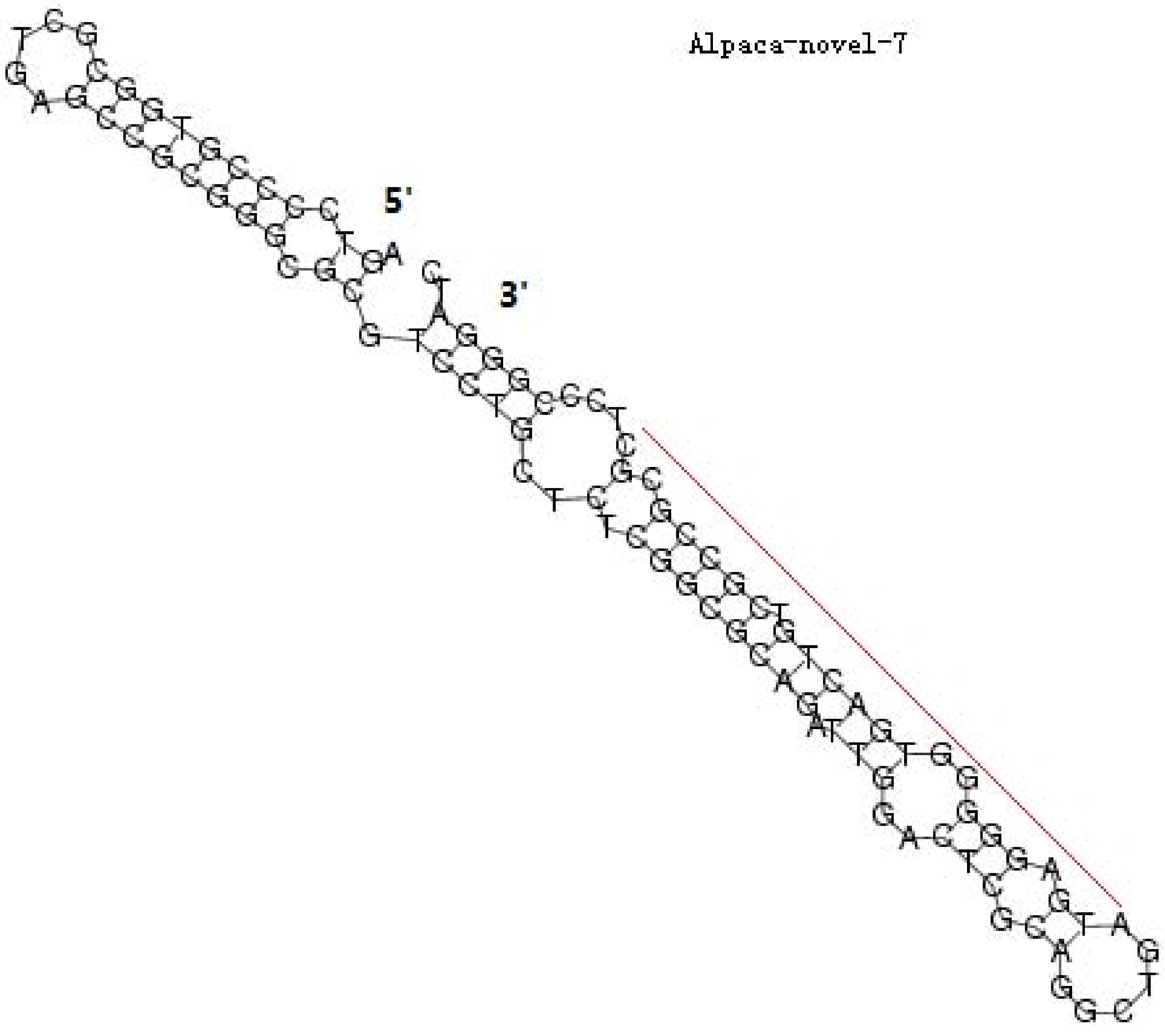
Alpaca-novel-7
5'
3'

Alpaca-novel-8

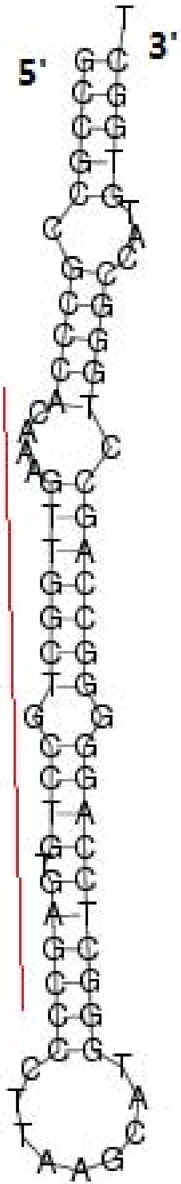

Alpaca-novel-9

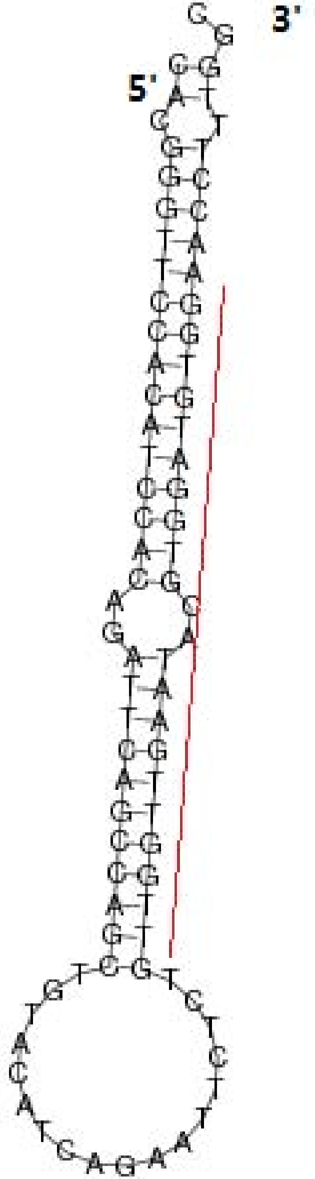

Alpaca-novel-10

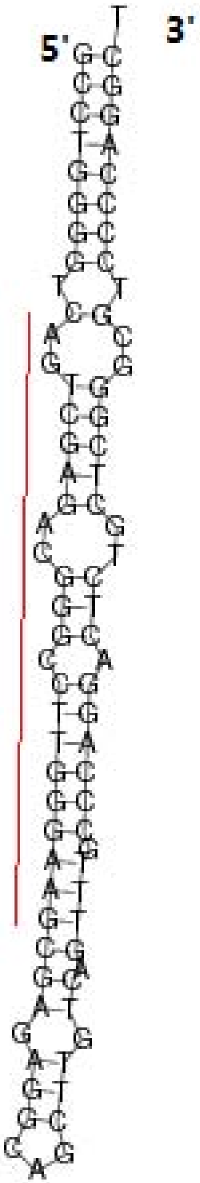

Alpaca-novel-11

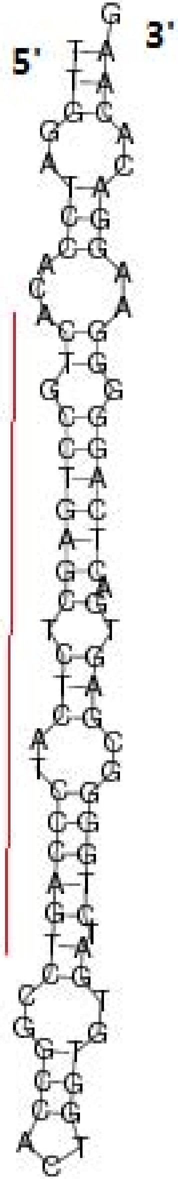

Alpaca-novel-12

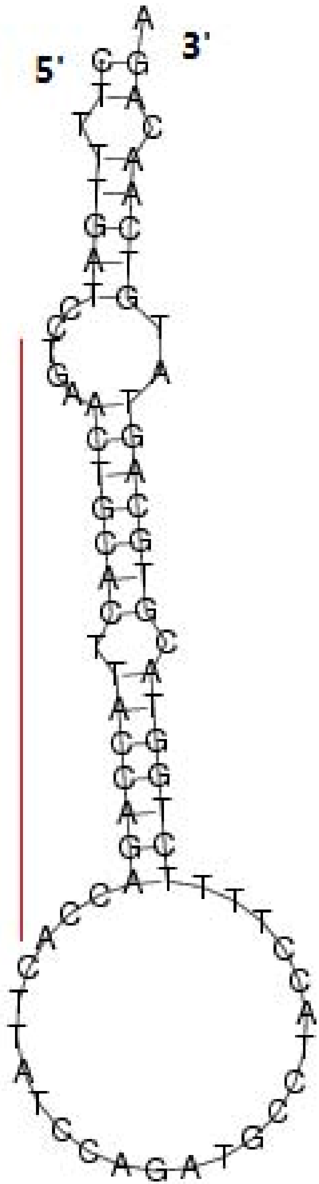

Alpaca-novel-13

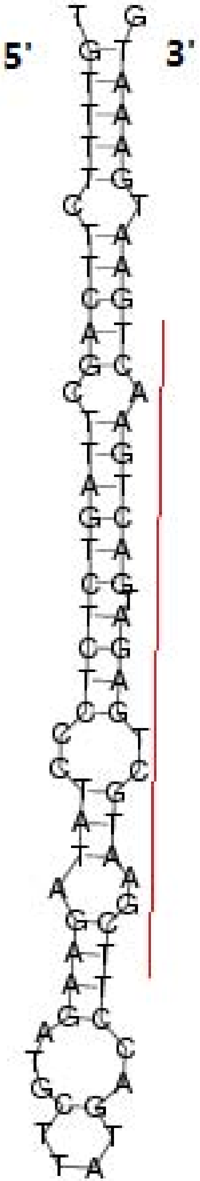

Alpaca-novel-14

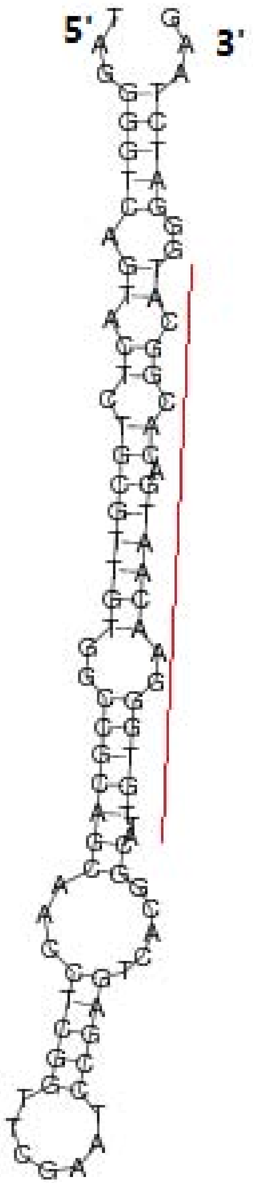

Alpaca-novel-15

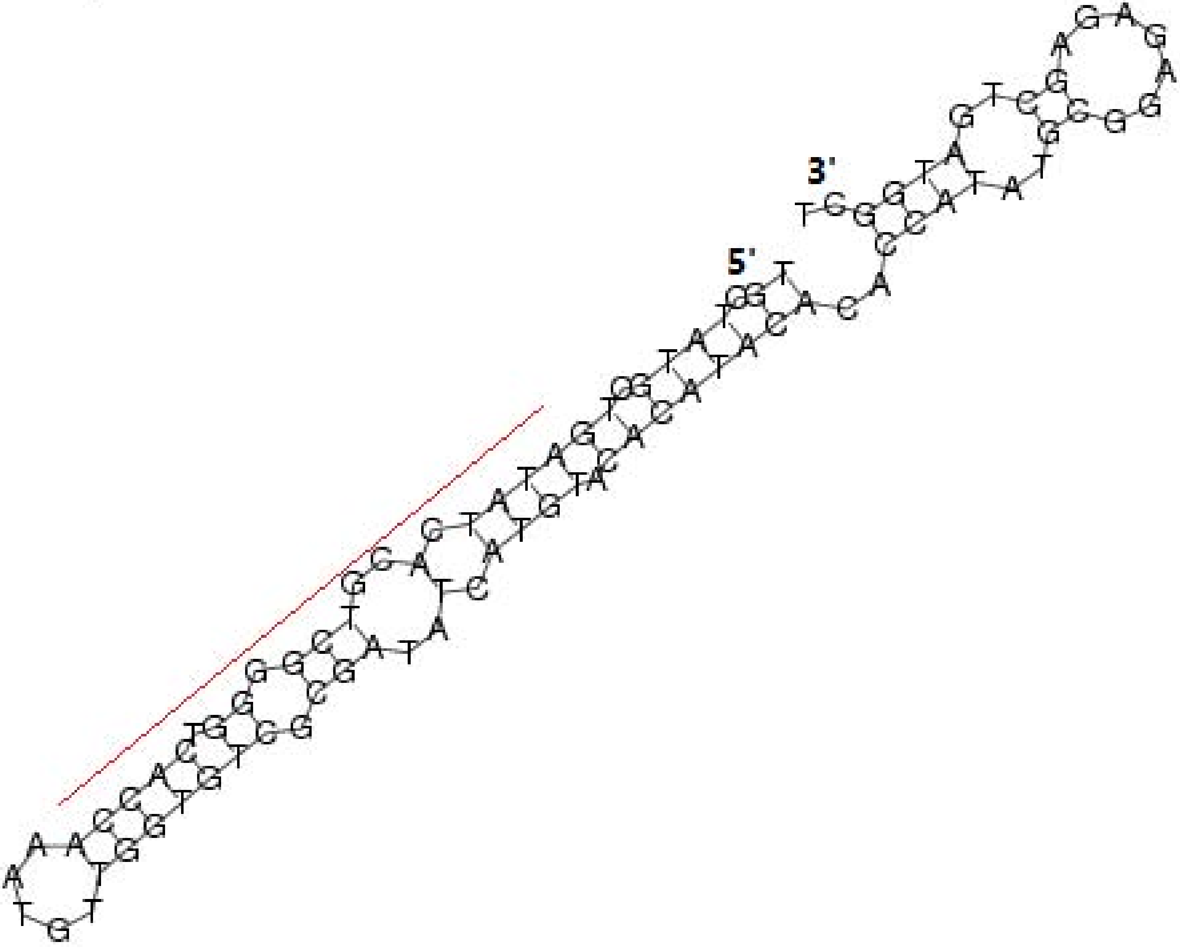

Alpaca-novel-16

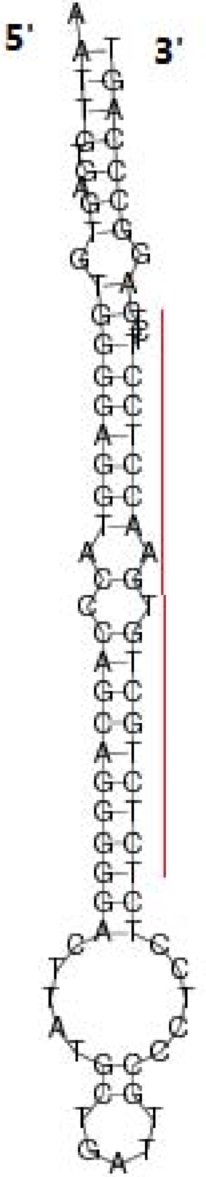

Alpaca-novel-17

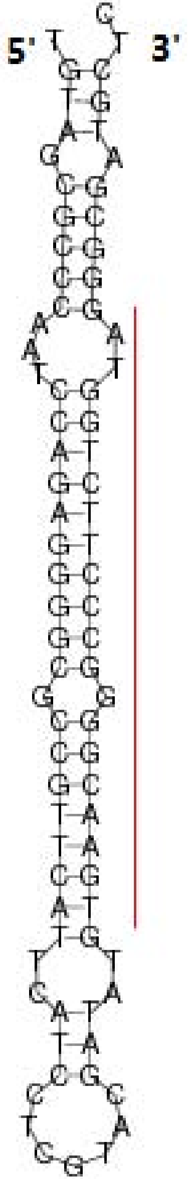

Alpaca-novel-18

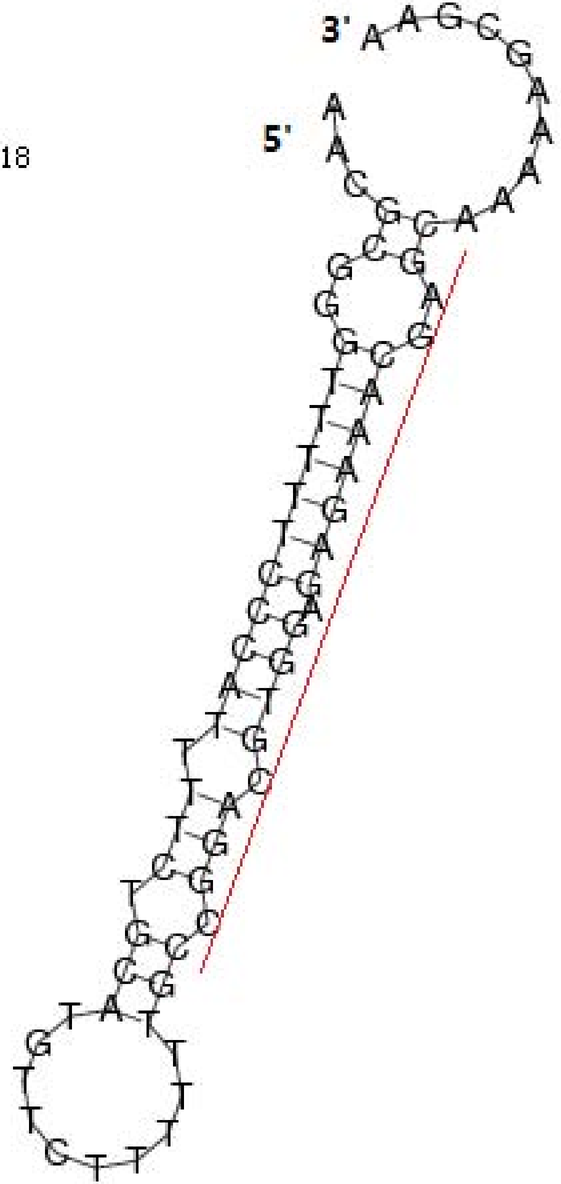

Alpaca-novel-19

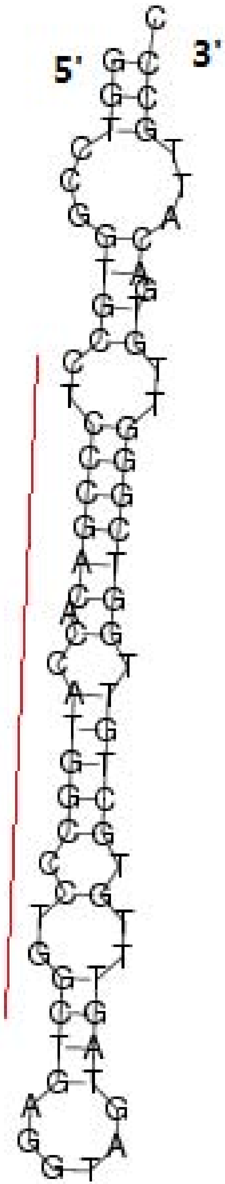

Alpaca-novel-20

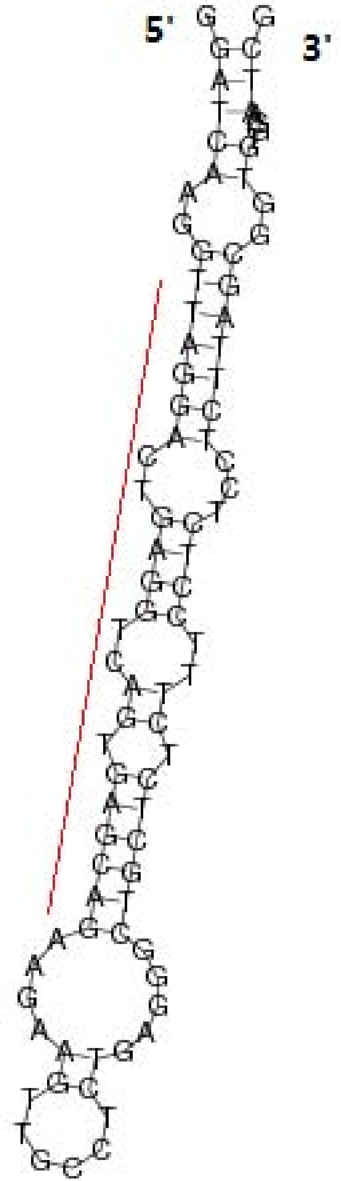

Alpaca-novel-21

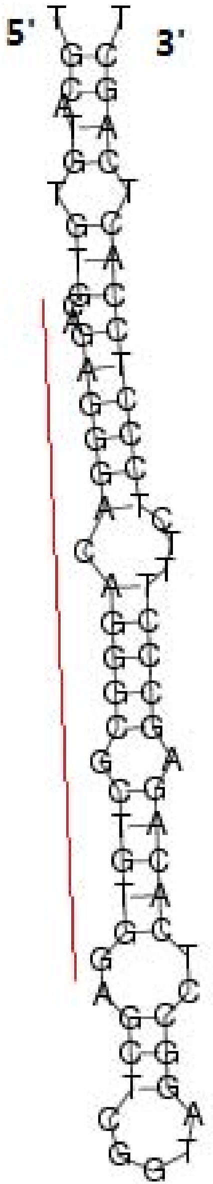

Alpaca-novel-22

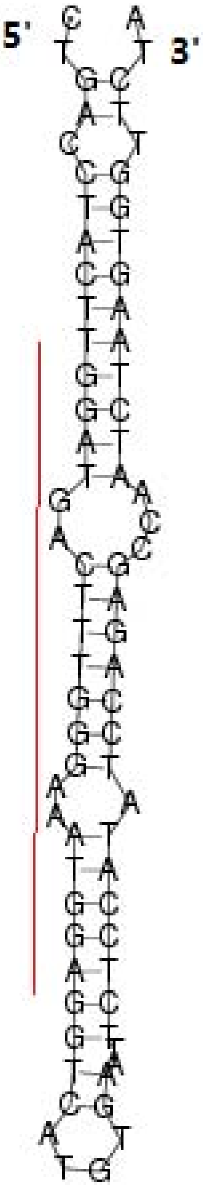

Alpaca-novel-23

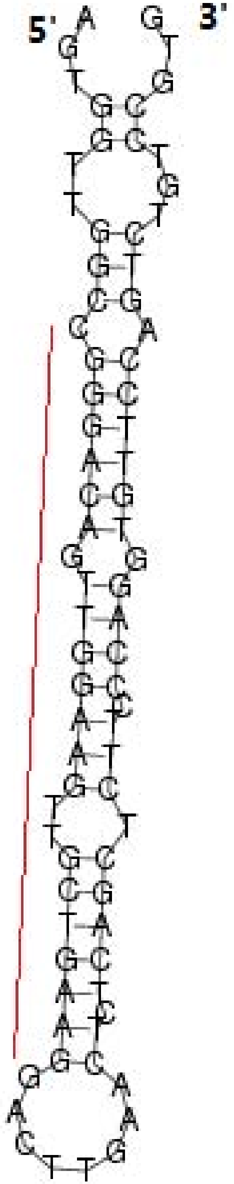

Alpaca-novel-24

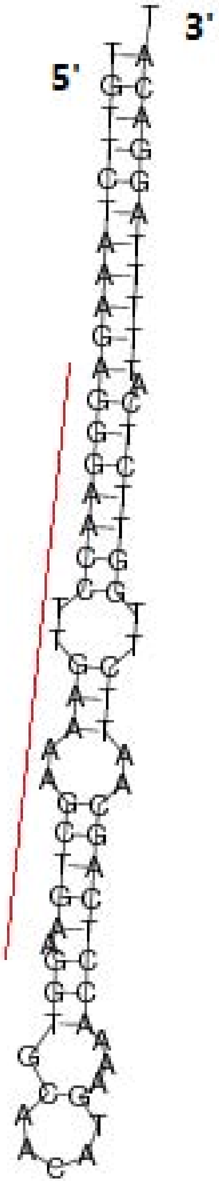

Alpaca-novel-25

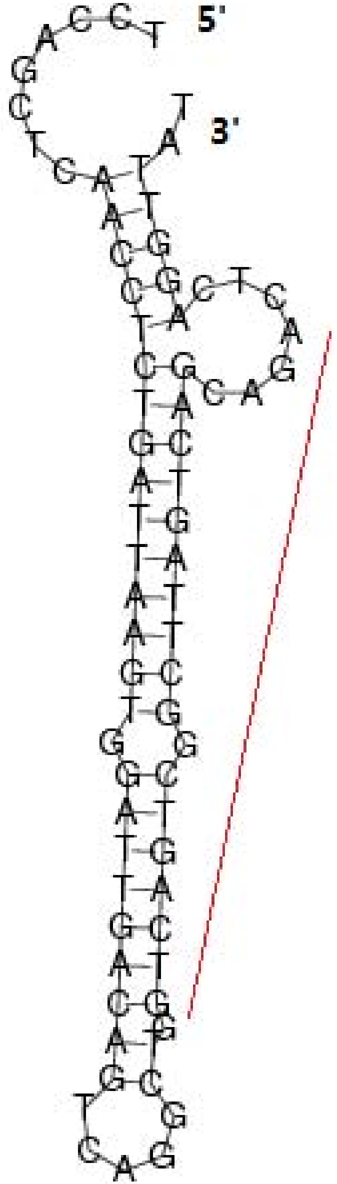

Alpaca-novel-26

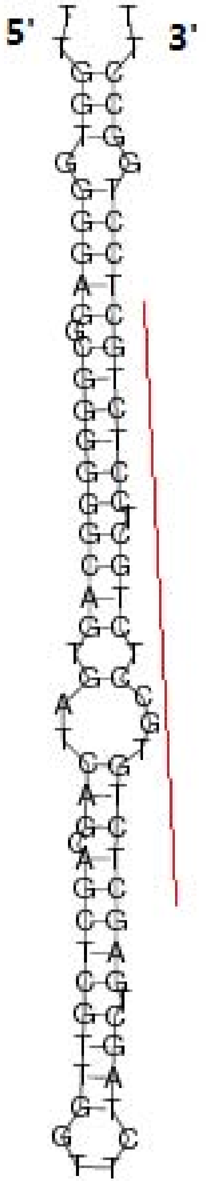

Alpaca-novel-27

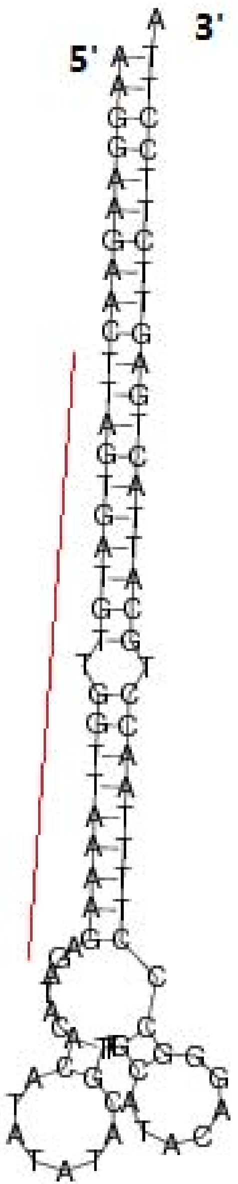

Alpaca-novel-28

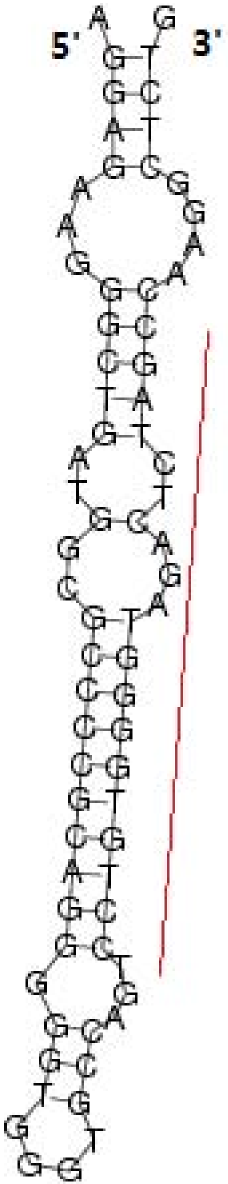

Alpaca-novel-29

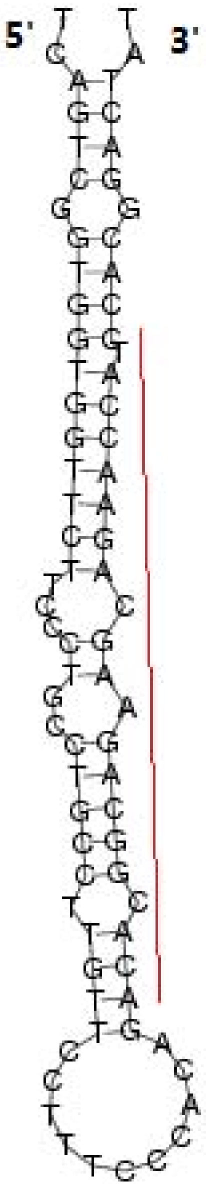

Alpaca-novel-30

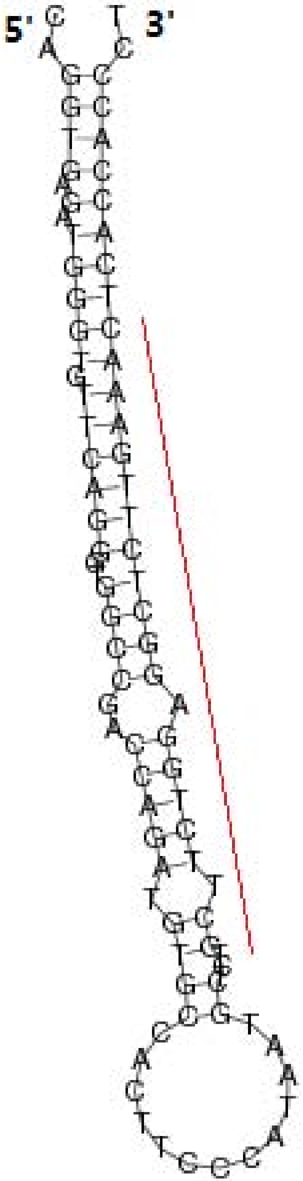

Alpaca-novel-31

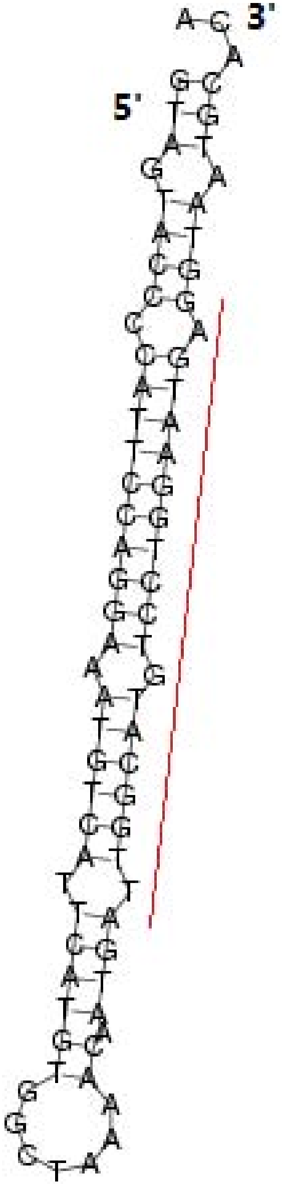

Alpaca-novel-32

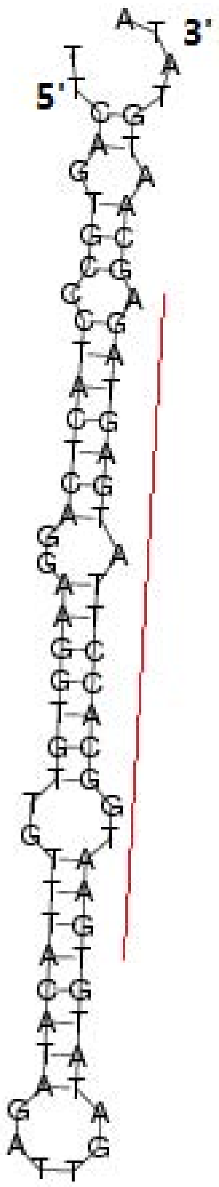

Alpaca-novel-33

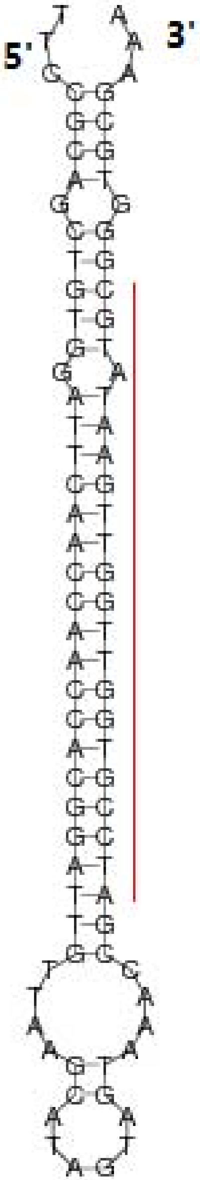

Alpaca-novel-34

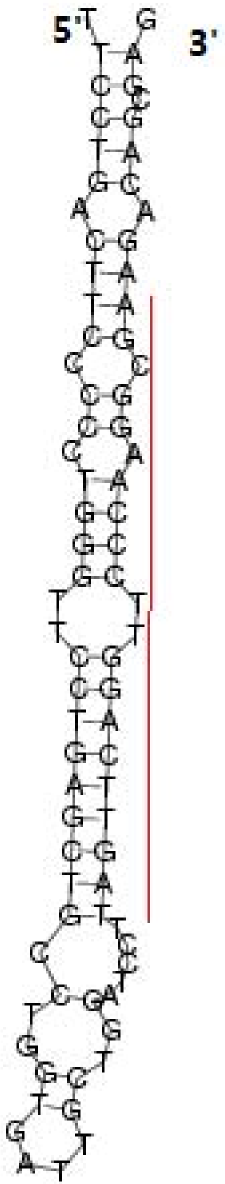

Alpaca-novel-35

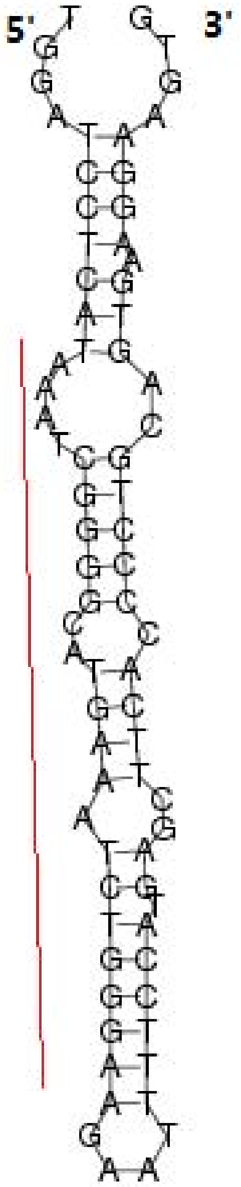

Alpaca-novel-36

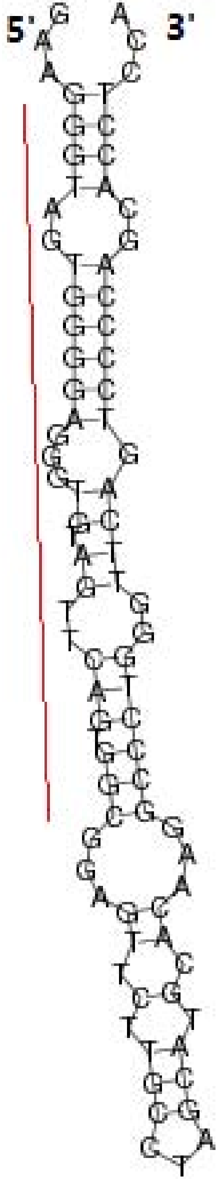

Alpaca-novel-37

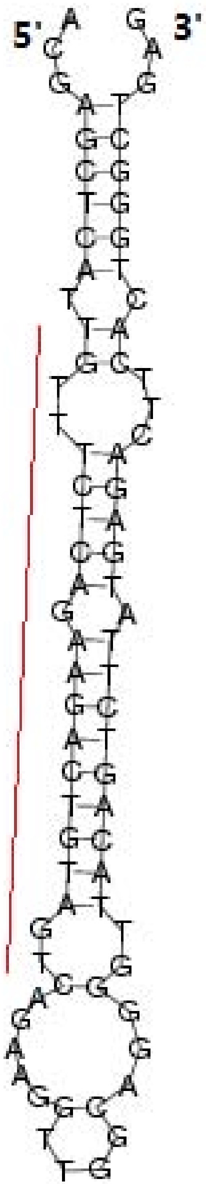

Alpaca-novel-38

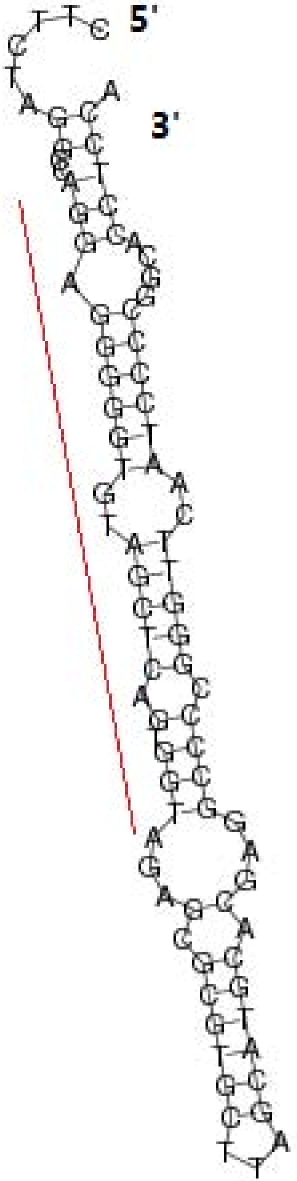

Alpaca-novel-39

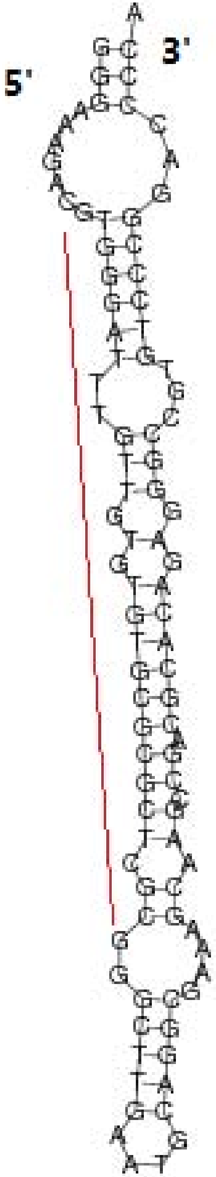

Alpaca-novel-40

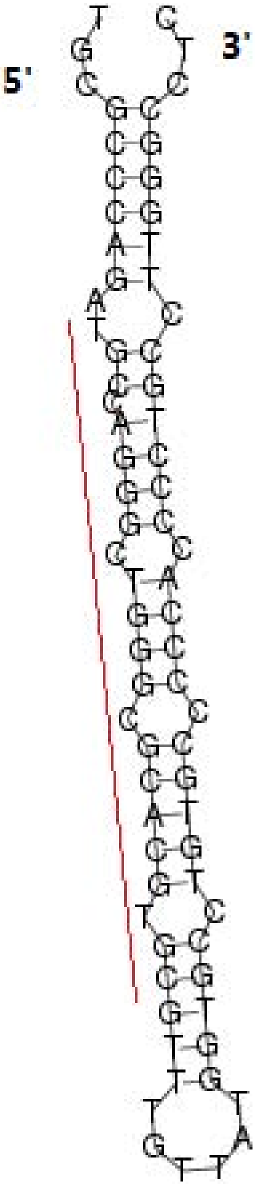

Alpaca-novel-41

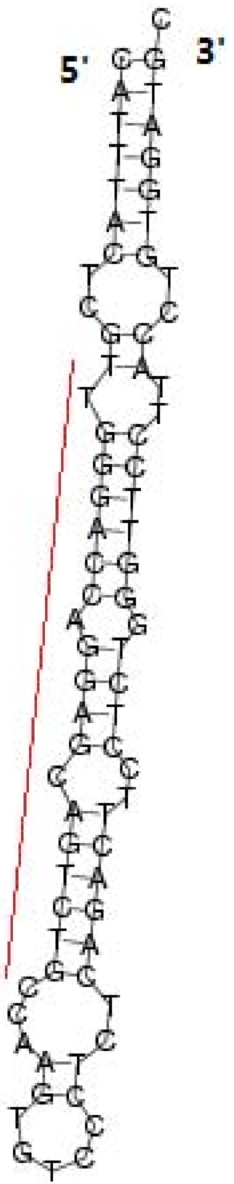

Alpaca-novel-42

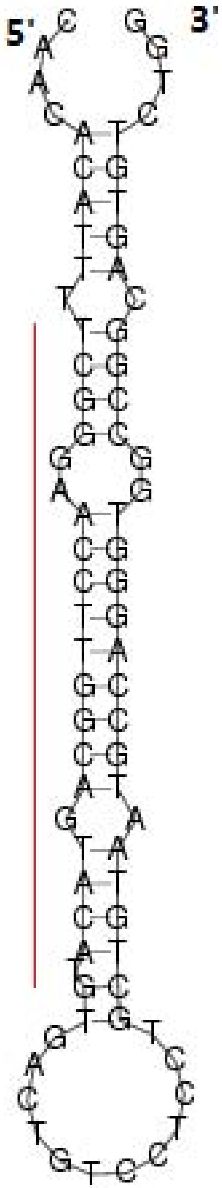

Alpaca-novel-43

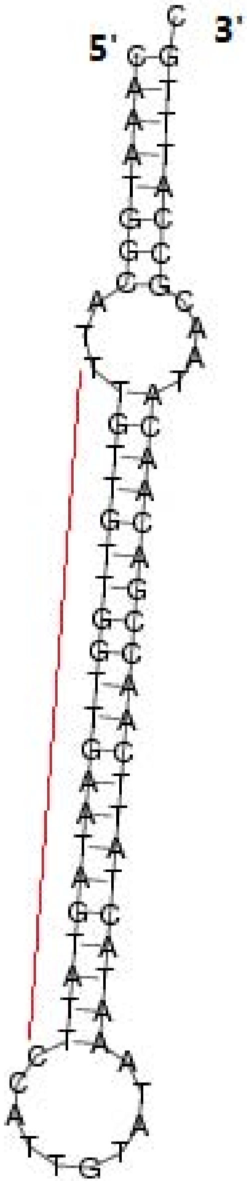

Alpaca-novel-44

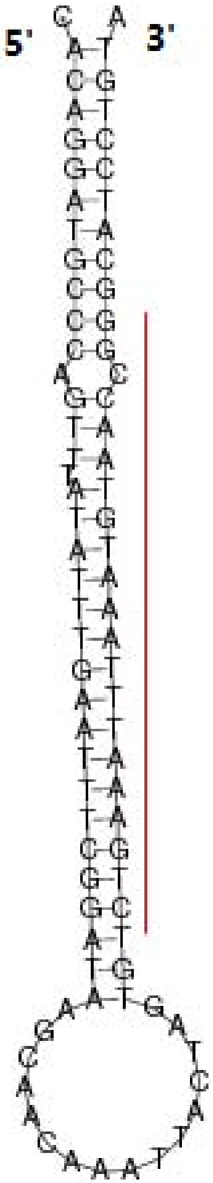

Alpaca-novel-45

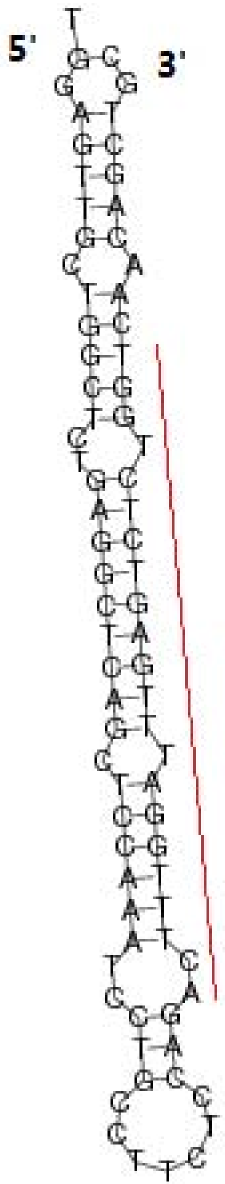

Alpaca-novel-46

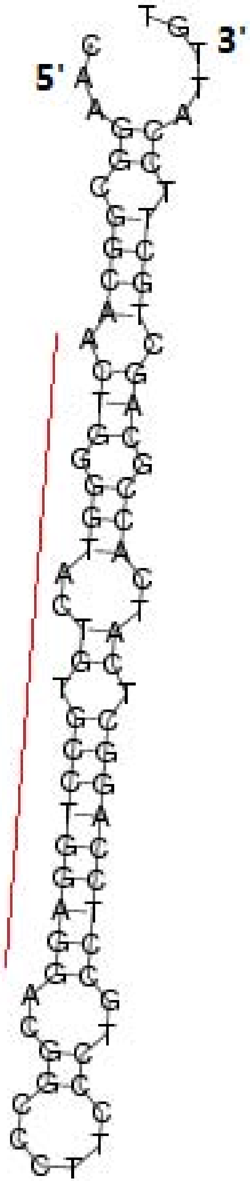

Alpaca-novel-47

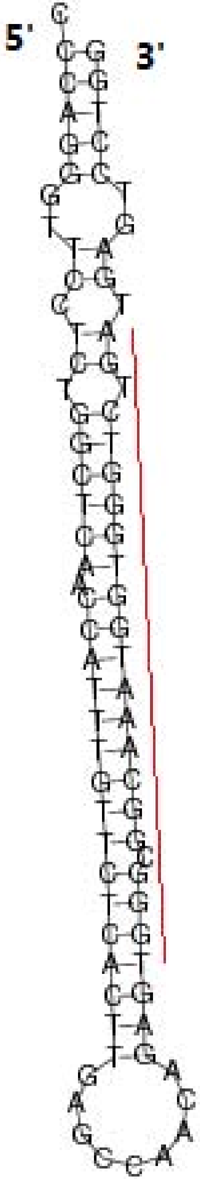

Alpaca-novel-48

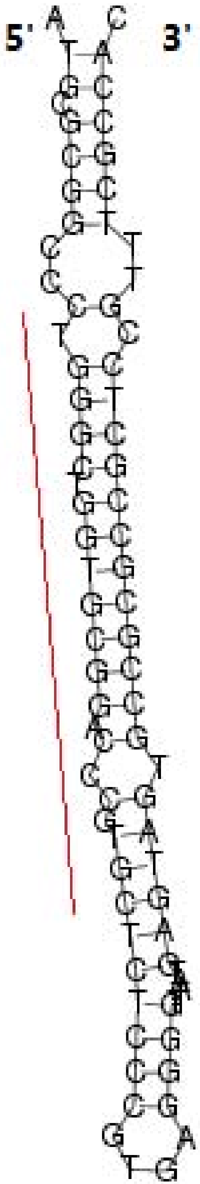

Alpaca-novel-49

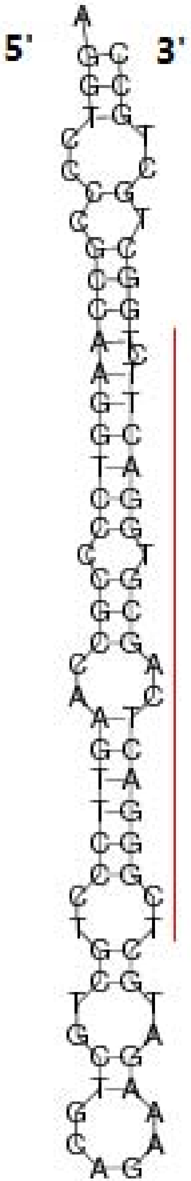

Alpaca-novel-50

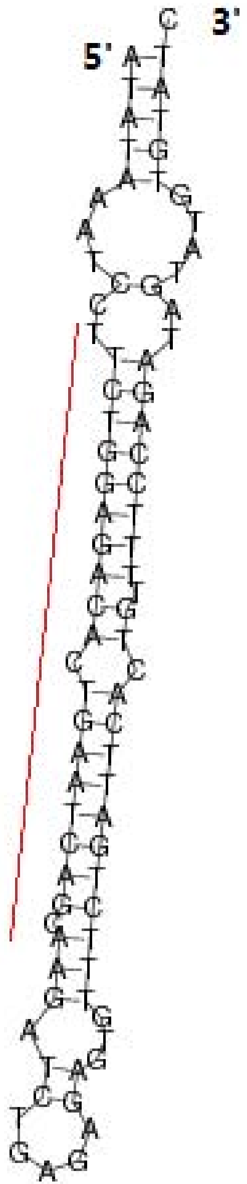

Alpaca-novel-51

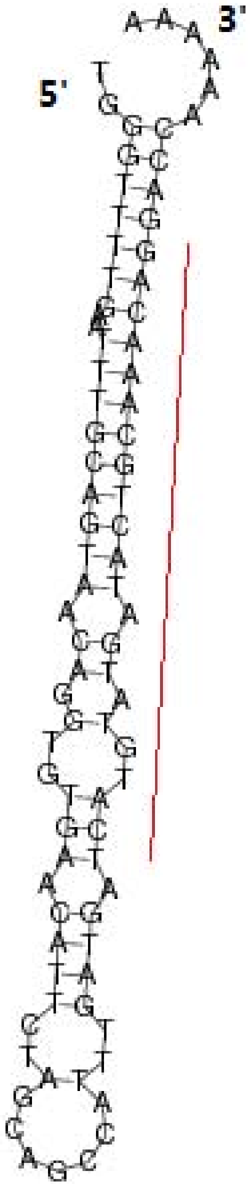

Alpaca-novel-52

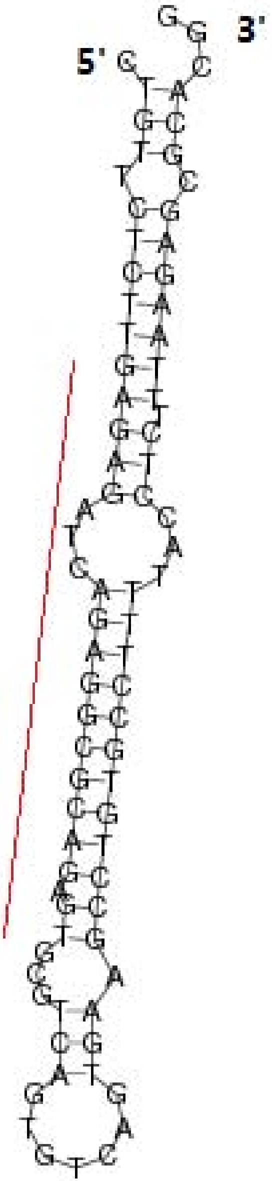

Alpaca-novel-53

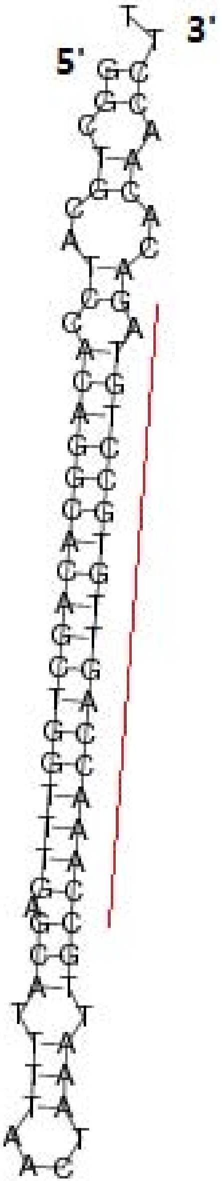

Alpaca-novel-54

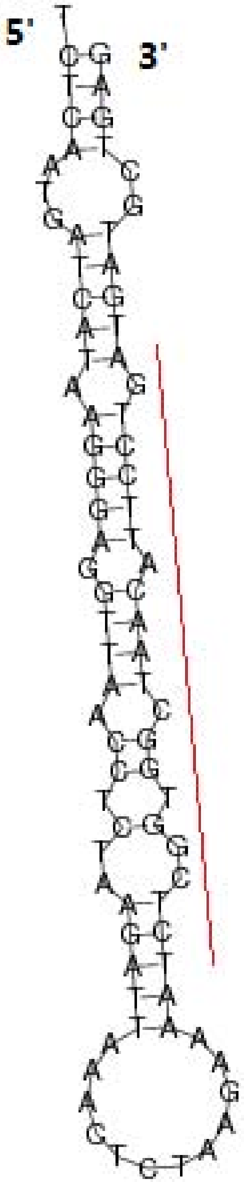

Alpaca-novel-55

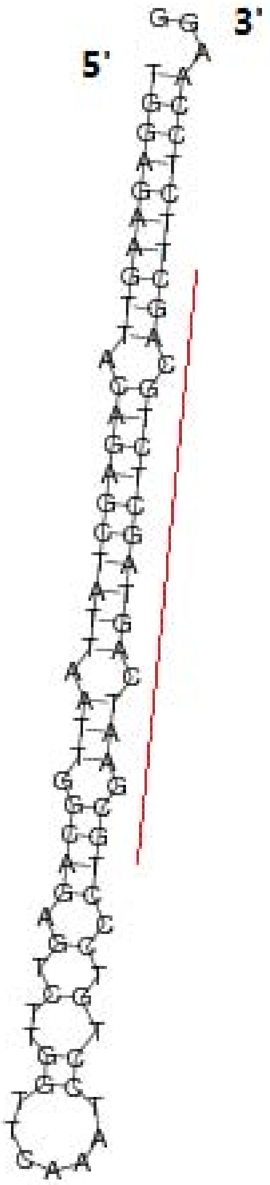

Alpaca-novel-56

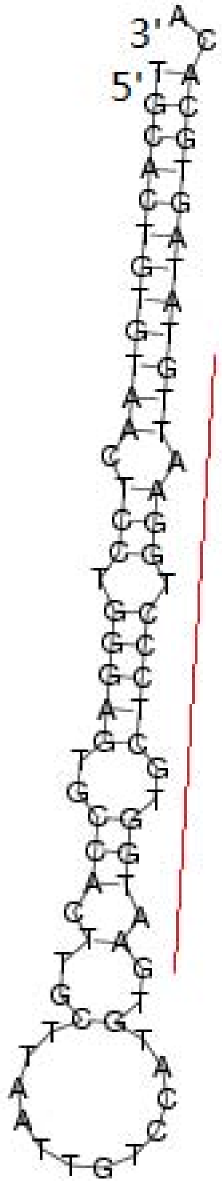

Alpaca-novel-57

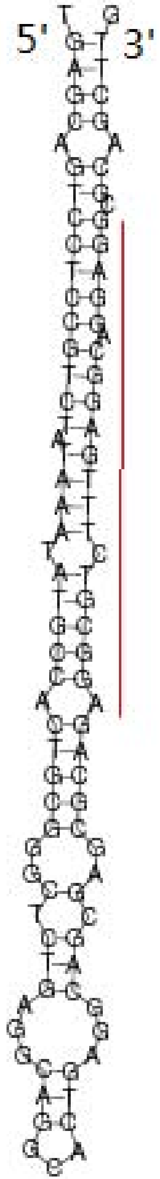

Alpaca-novel-58

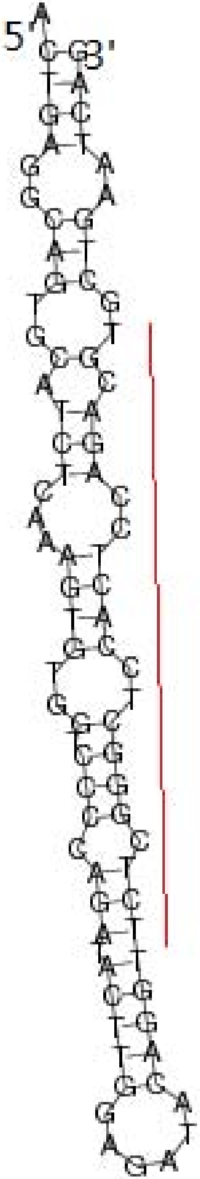

Alpaca-novel-59

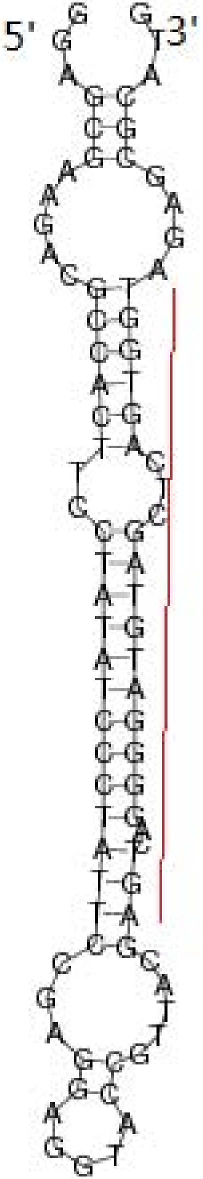

Alpaca-novel-60

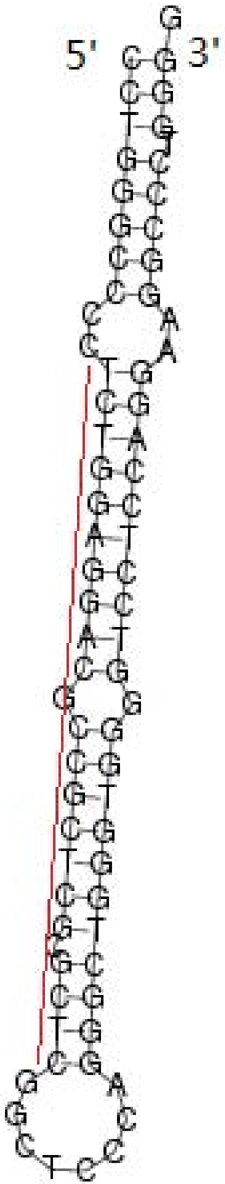

Alpaca-novel-61

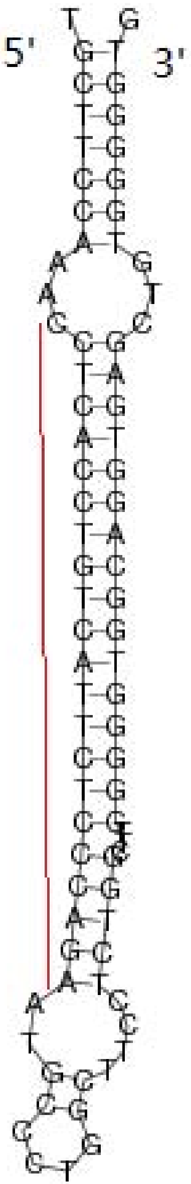

Alpaca-novel-62

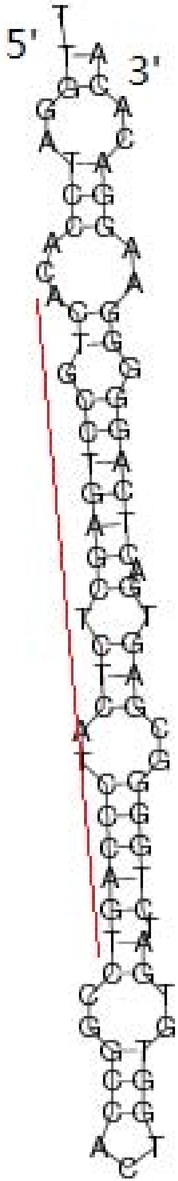

Alpaca-novel-63

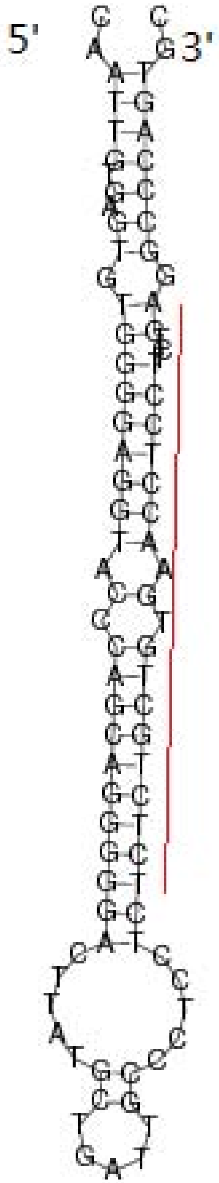

Alpaca-novel-64

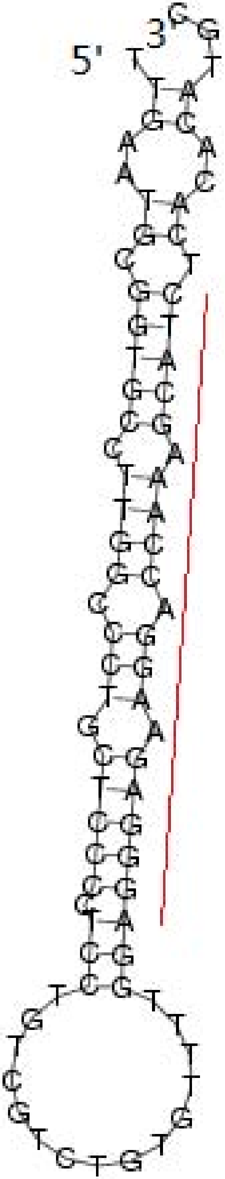

Alpaca-novel-65

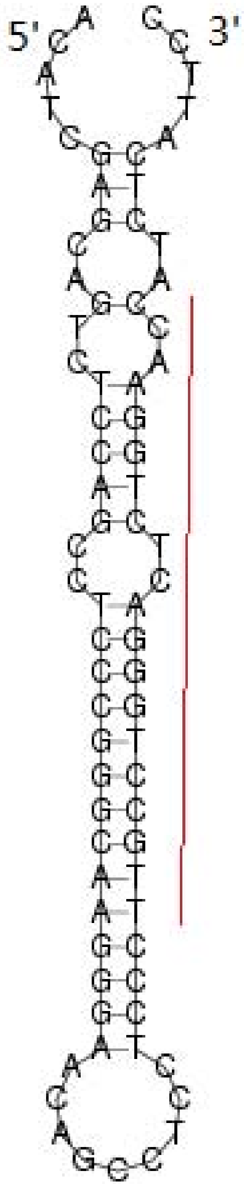

Alpaca-novel-66

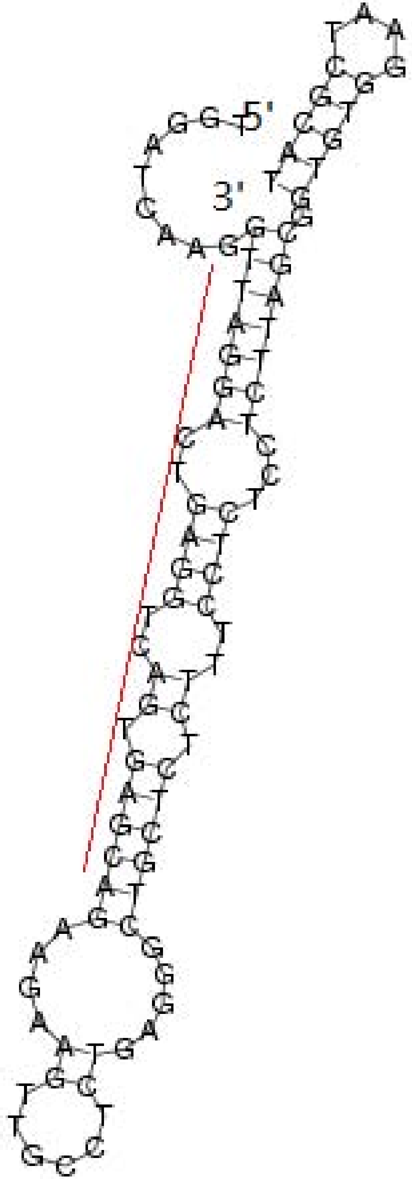

Alpaca-novel-67

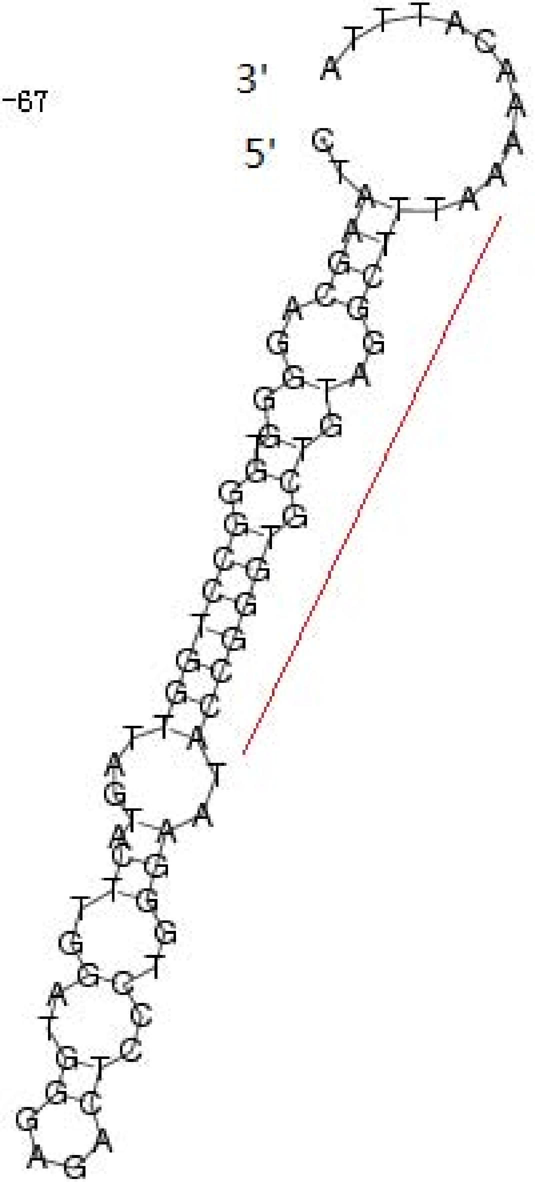

Alpaca-novel-68

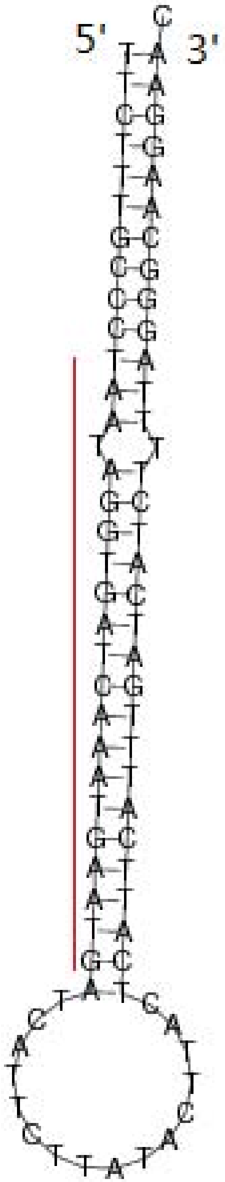

Alpaca-novel-69

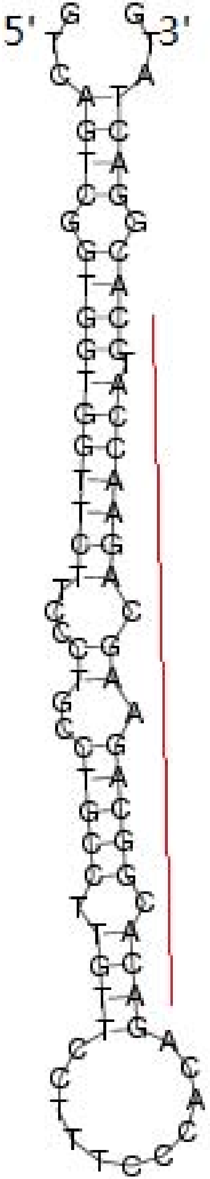

Alpaca-novel-70

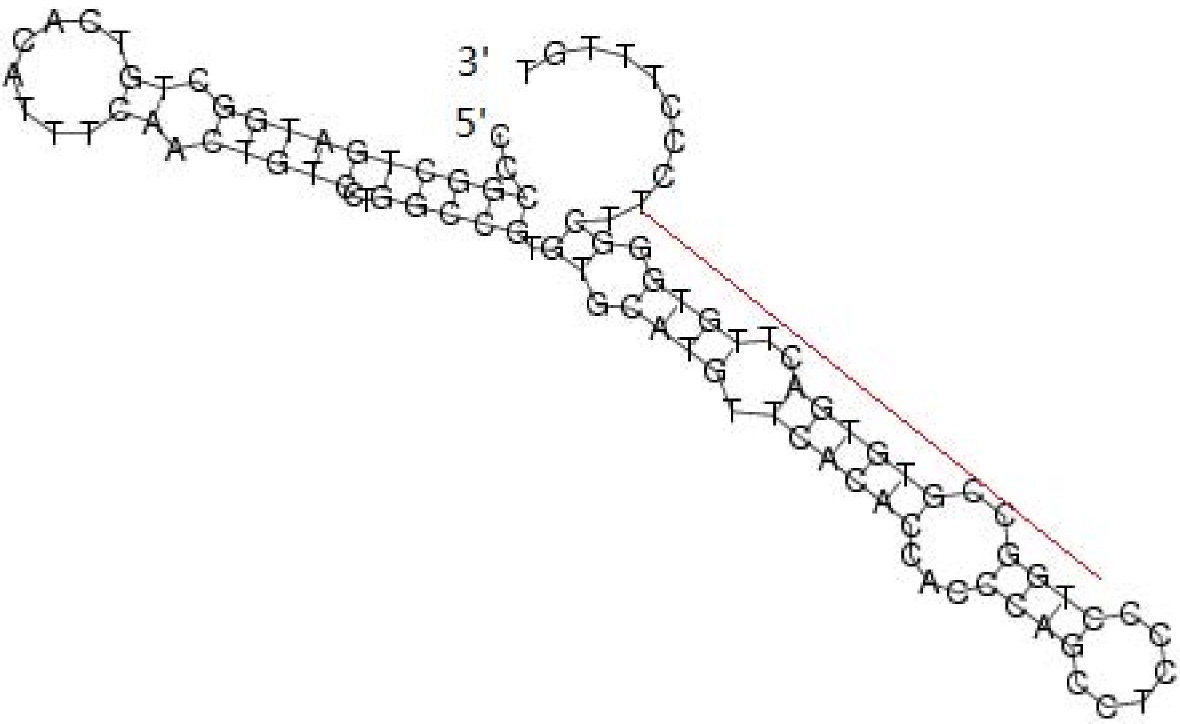

Alpaca-novel-71

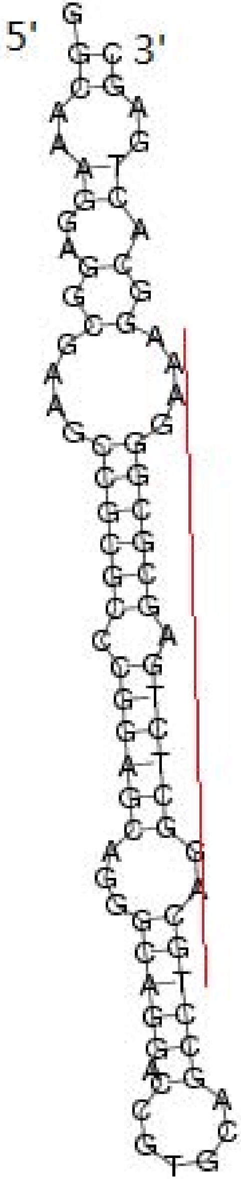

Alpaca-novel-72

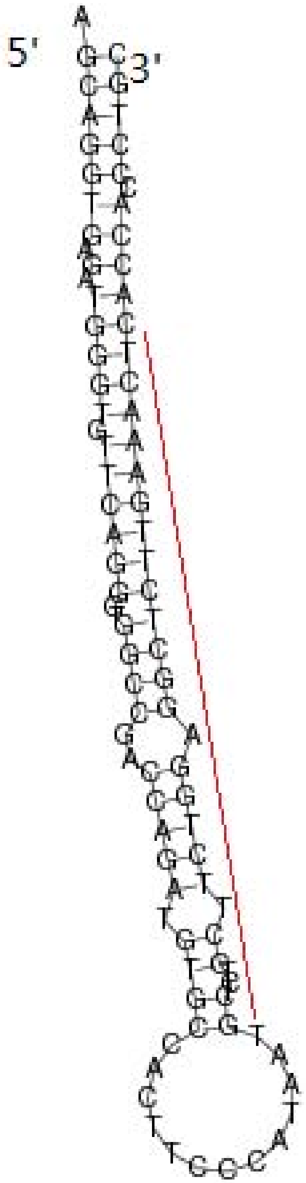

Alpaca-novel-73

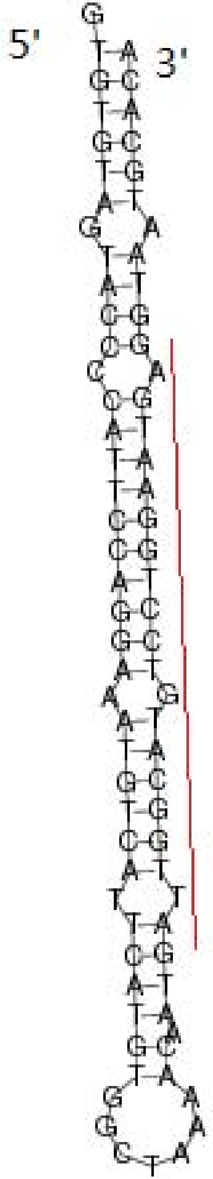

Alpaca-novel-74

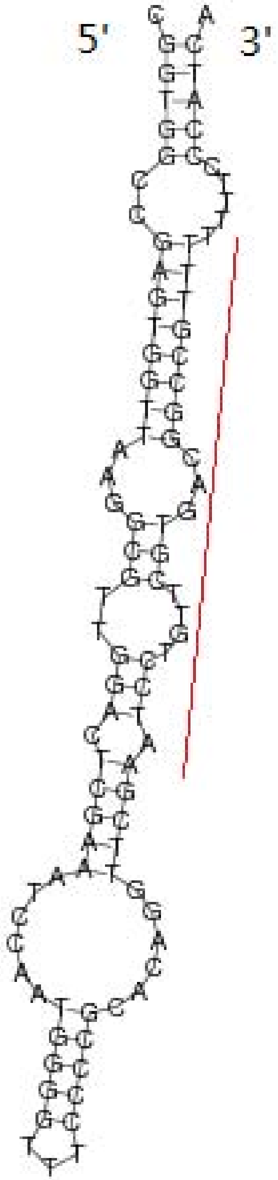

Alpaca-novel-75

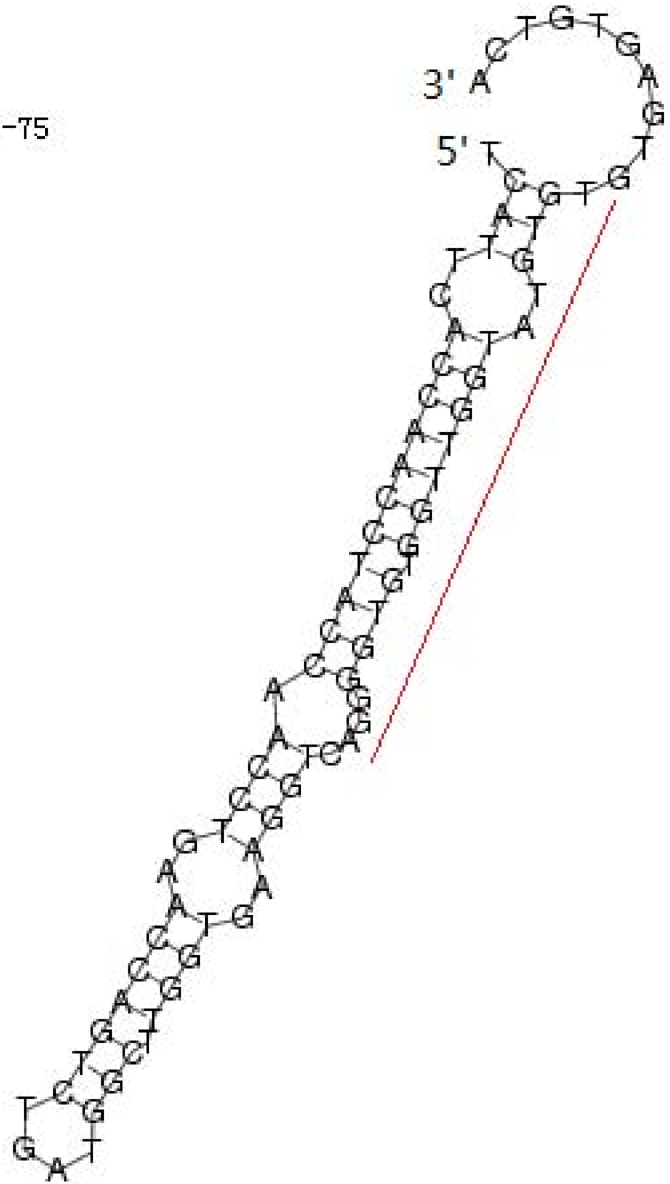

Alpaca-novel-76

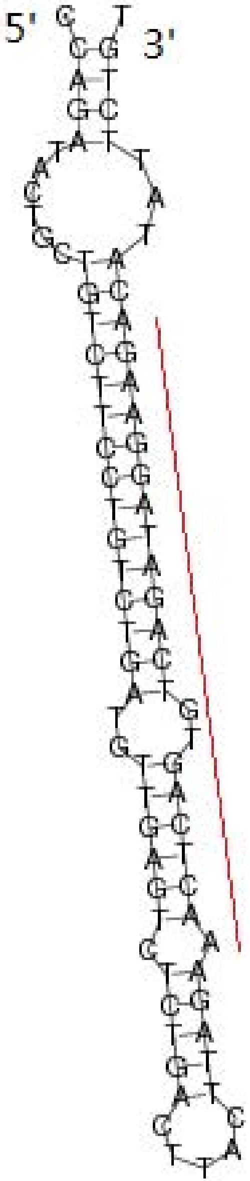

Alpaca-novel-77

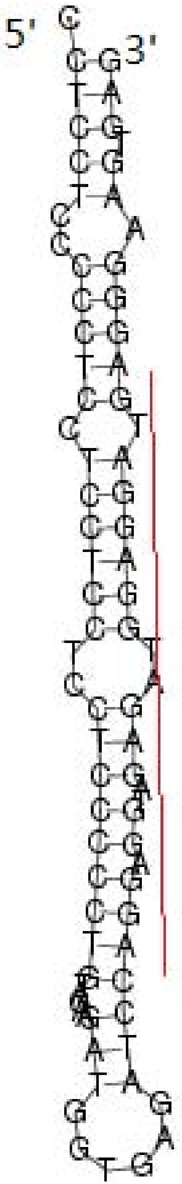

Alpaca-novel-78

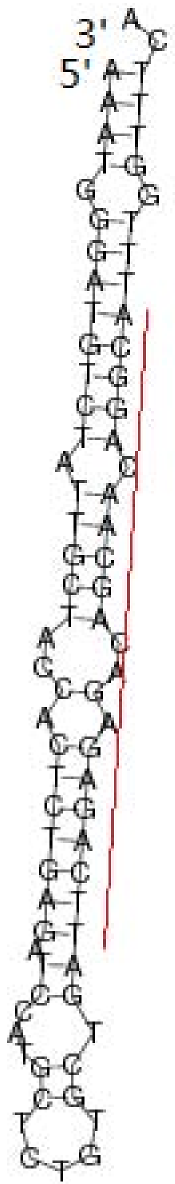

Alpaca-novel-79

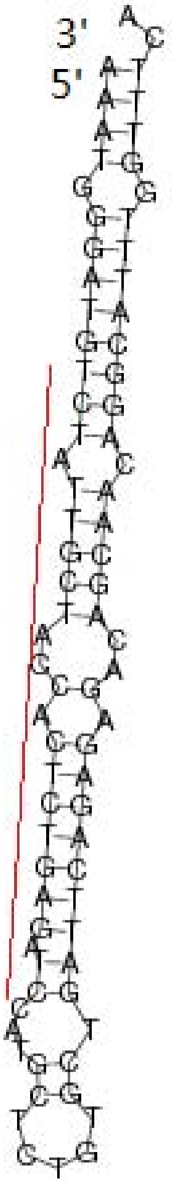

Alpaca-novel-80

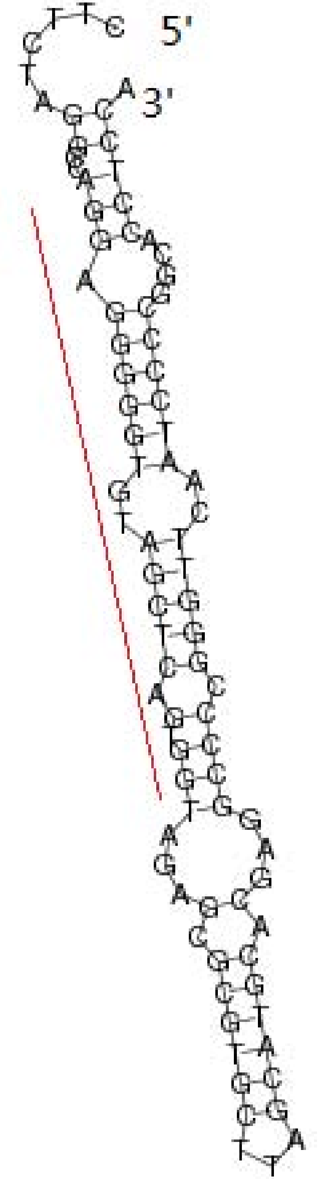

Alpaca-novel-81

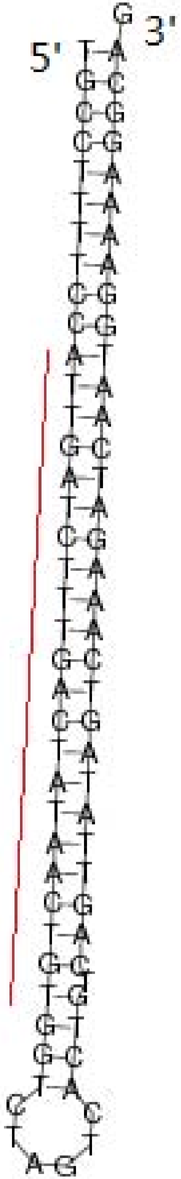

Alpaca-novel-82

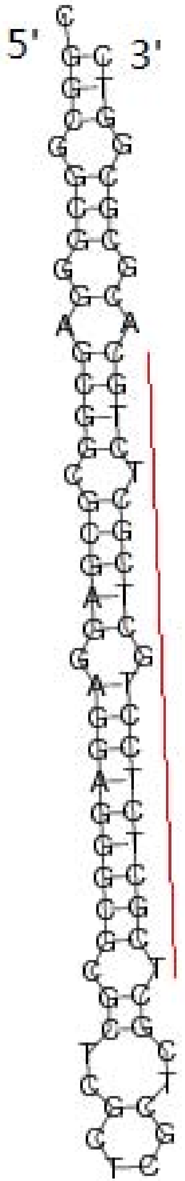

Alpaca-novel-83

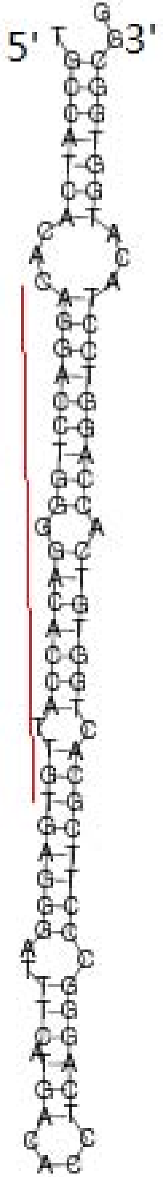

Alpaca-novel-84

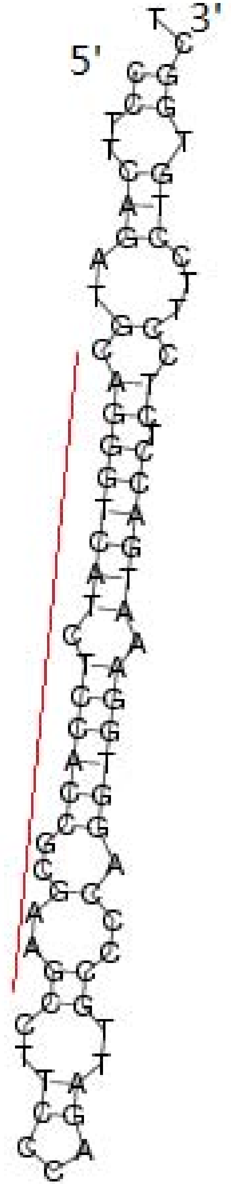

Alpaca-novel-85

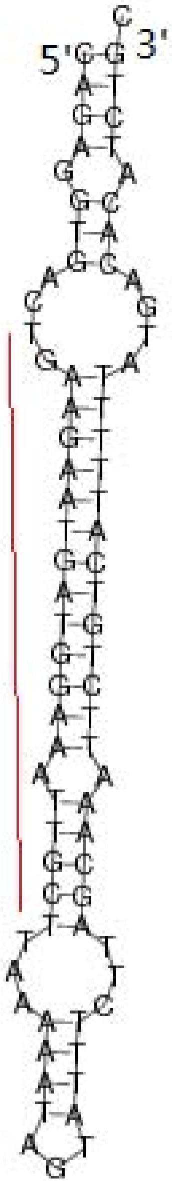

Alpaca-novel-86

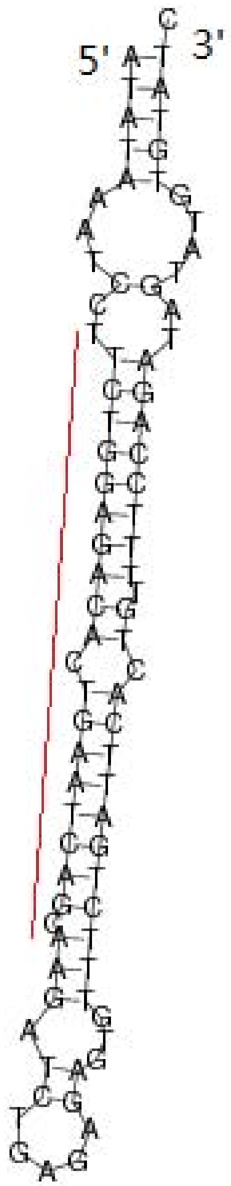

Alpaca-novel-87

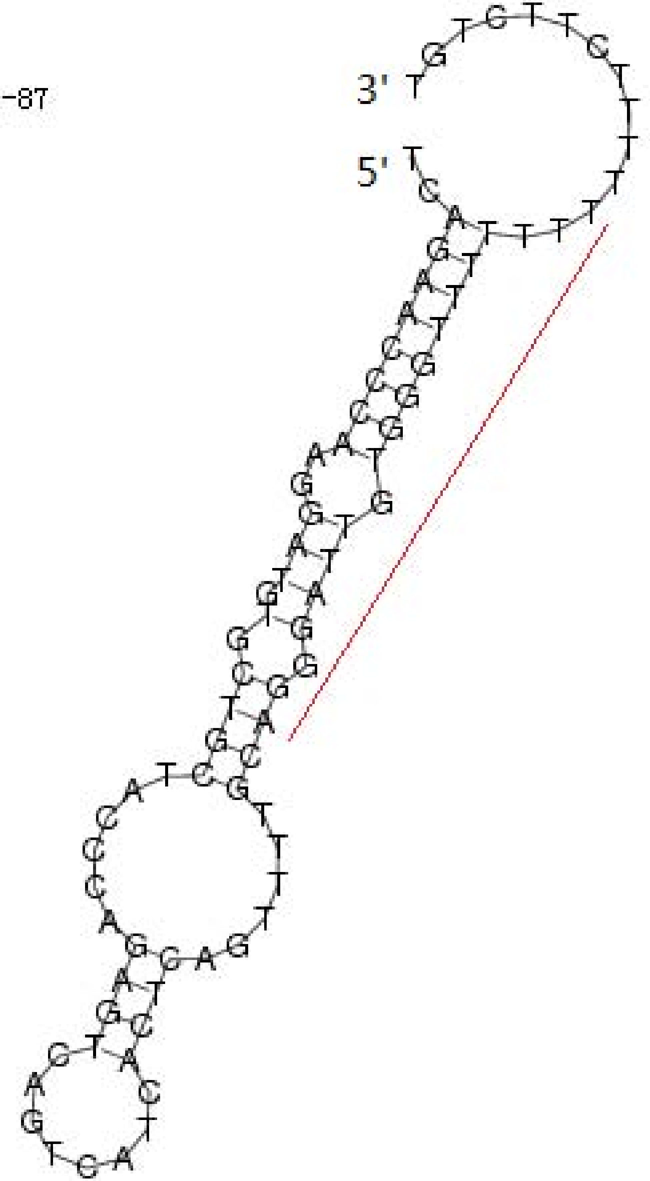

Supplement: Additional file 4 — Structure S4. Stem–loop structure of novel miRNAs. The stem-loop structure of 87 novel miRNAs in white alpaca and brown alpaca. The red underlined sequences are the predicted mature sequences. [file 1471-2164-13-555-S4.pdf]
